# A computational ontology framework for the synthesis of multi-level pathology reports from brain MRI scans

**DOI:** 10.1177/13872877251331222

**Published:** 2025-04-21

**Authors:** Devesh Singh, Alice Grazia, Achim Reiz, Andreas Hermann, Slawek Altenstein, Lukas Beichert, Alexander Bernhardt, Katharina Buerger, Michaela Butryn, Peter Dechent, Emrah Duezel, Michael Ewers, Klaus Fliessbach, Silka D Freiesleben, Wenzel Glanz, Stefan Hetzer, Daniel Janowitz, Ingo Kilimann, Okka Kimmich, Christoph Laske, Johannes Levin, Andrea Lohse, Falk Luesebrink, Matthias Munk, Robert Perneczky, Oliver Peters, Lukas Preis, Josef Priller, Johannes Prudlo, Boris S Rauchmann, Ayda Rostamzadeh, Nina Roy-Kluth, Klaus Scheffler, Anja Schneider, Luisa S Schneider, Björn H Schott, Annika Spottke, Eike J Spruth, Matthis Synofzik, Jens Wiltfang, Frank Jessen, Stefan J Teipel, Martin Dyrba, ADNI, AIBL, FTLDNI, study groups

**Affiliations:** 1 German Center for Neurodegenerative Diseases (DZNE), Rostock/Greifswald, Germany; 2 Chair of Business Information Systems, Rostock University, Rostock, Germany; 3 Section for Translational Neurodegeneration Albrecht Kossel, Department of Neurology, University Hospital Rostock, Rostock, Germany; 4 German Center for Neurodegenerative Diseases (DZNE), Berlin, Germany; 5 Department of Psychiatry and Psychotherapy, Charité – University Medicine Berlin, Berlin, Germany; 6 Division Translational Genomics of Neurodegenerative Diseases, Hertie Institute for Clinical Brain Research and Center of Neurology, University of Tübingen, Tübingen, Germany; 7 German Center for Neurodegenerative Diseases (DZNE), Munich, Germany; 8 Department of Neurology, University Hospital of Munich, Ludwig-Maximilians-Universität (LMU) Munich, Munich, Germany; 9 Institute for Stroke and Dementia Research, LMU Munich University Hospital, Munich, Germany; 10 German Center for Neurodegenerative Diseases (DZNE), Magdeburg, Germany; 11 Institute for Cognitive Neurology and Dementia Research, Faculty of Medicine, University Hospital Magdeburg, Magdeburg, Germany; 12 MR-Research in Neurosciences, Department of Cognitive Neurology, Georg-August-University Goettingen, Goettingen, Germany; 13 German Center for Neurodegenerative Diseases (DZNE), Bonn, Germany; 14 Department for Neurodegenerative Diseases and Gerontopsychiatry, University of Bonn, Bonn, Germany; 15 Berlin Center for Advanced Neuroimaging, Charité University Medicine Berlin, Berlin, Germany; 16 Department of Psychosomatic Medicine, Rostock University Medical Center, Rostock, Germany; 17 German Center for Neurodegenerative Diseases (DZNE), Tübingen, Germany; 18 Section for Dementia Research, Hertie Institute for Clinical Brain Research, Department of Psychiatry and Psychotherapy, University Hospital Tübingen, Tübingen, Germany; 19 Munich Cluster for Systems Neurology (SyNergy), Munich, Germany; 20 Department of Psychiatry and Psychotherapy, University Hospital Tübingen, Tübingen, Germany; 21 Department of Psychiatry and Psychotherapy, University Hospital, LMU Munich, Munich, Germany; 22 Ageing Epidemiology Research Unit, School of Public Health, Faculty of Medicine, Imperial College London, London, UK; 23 Department of Psychiatry and Psychotherapy, School of Medicine, Technical University of Munich,Munich, Germany; 24 UK Dementia Research Institute, University of Edinburgh, Edinburgh, UK; 25 Department of Neurology, University Medical Centre, Rostock, Germany; 26 Sheffield Institute for Translational Neuroscience, The University of Sheffield, Sheffield, UK; 27 Department of Neuroradiology, University Hospital, LMU Munich, Germany; 28 Department of Psychiatry, Medical Faculty, University of Cologne, Cologne, Germany; 29 Department for Biomedical Magnetic Resonance, University of Tübingen, Tübingen, Germany; 30 German Center for Neurodegenerative Diseases (DZNE), Goettingen, Germany; 31 Department of Psychiatry and Psychotherapy, University Medical Center Goettingen, Goettingen, Germany; 32 Leibniz Institute for Neurobiology (LG), Magdeburg, Germany; 33 Department of Neurology, University Hospital Bonn, Bonn, Germany; 34 Neurosciences and Signaling Group, Institute of Biomedicine (iBiMED), Department of Medical Sciences, University of Aveiro, Aveiro, Portugal; 35 Cologne Excellence Cluster on Cellular Stress Responses in Aging-Associated Diseases, Faculty of Medicine, University of Cologne, Cologne, Germany

**Keywords:** Alzheimer's disease, brain volumetry, computer graphics, frontotemporal dementia, magnetic resonance imaging, neuroanatomy, ontology

## Abstract

**Background:**

Convolutional neural network (CNN) based volumetry of MRI data can help differentiate Alzheimer's disease (AD) and the behavioral variant of frontotemporal dementia (bvFTD) as causes of cognitive decline and dementia. However, existing CNN-based MRI volumetry tools lack a structured hierarchical representation of brain anatomy, which would allow for aggregating regional pathological information and automated computational inference.

**Objective:**

Develop a computational ontology pipeline for quantifying hierarchical pathological abnormalities and visualize summary charts for brain atrophy findings, aiding differential diagnosis.

**Methods:**

Using FastSurfer, we segmented brain regions and measured volume and cortical thickness from MRI scans pooled across multiple cohorts (N = 3433; ADNI, AIBL, DELCODE, DESCRIBE, EDSD, and NIFD), including healthy controls, prodromal and clinical AD cases, and bvFTD cases. Employing the Web Ontology Language (OWL), we built a semantic model encoding hierarchical anatomical information. Additionally, we created summary visualizations based on sunburst plots for visual inspection of the information stored in the ontology.

**Results:**

Our computational framework dynamically estimated and aggregated regional pathological deviations across different levels of neuroanatomy abstraction. The disease similarity index derived from the volumetric and cortical thickness deviations achieved an AUC of 0.88 for separating AD and bvFTD, which was also reflected by distinct atrophy profile visualizations.

**Conclusions:**

The proposed automated pipeline facilitates visual comparison of atrophy profiles across various disease types and stages. It provides a generalizable computational framework for summarizing pathologic findings, potentially enhancing the physicians’ ability to evaluate brain pathologies robustly and interpretably.

## Introduction

Alzheimer's disease (AD) is the primary cause of dementia, contributing to over two-thirds of all dementia cases. There are different pathological neuronal mechanisms associated with AD neurodegeneration,^1,2^ leading to regional volumetric changes such as gray matter volume reduction (atrophy) in the hippocampus, medial temporal lobe, and later in more widespread cortical areas. The decrease in volume and cortical thinning are identifiable structural abnormalities readily noticeable in T1-weighted MRI scans, apparent even during the early stages of AD.^3^ Due to the gradual nature of AD, it is difficult to diagnose its early stages clinically. The first symptomatic stage of AD is called mild cognitive impairment (MCI). It is reported that approximately one-third of the people with MCI will convert into dementia over a period of five years.^4^ It is important to note that MCI is a heterogeneous syndrome as it can be caused by several other underlying conditions, such as depression or vascular lesions.

There are also other neurological disorders that may resemble AD clinically. Frontotemporal lobar degeneration (FTLD) is a group of disorders, which are histopathologically distinct from AD. FTLD often occurs in patients under the age of 65 years and is a common cause of early-onset dementia.^5^ Clinically, there are three different subtypes of FTLD: the behavioral variant of frontotemporal dementia (bvFTD), semantic dementia (SD), and progressive nonfluent aphasia (PNFA). bvFTD accounts for approximately 40% of the FTLD cases and is generally marked by atrophy in the frontal cortex and the temporal poles, resulting in pronounced behavioral and personality changes.^6^ Notably, bvFTD may sometimes be hard to distinguish from AD due to overlapping patients’ age and affected cognitive domains.^7,8^

In recent years, there have been multiple studies on convolutional neural networks (CNNs) solving various medical imaging tasks. CNNs have also been applied to tasks like brain segmentation or identifying clinical stages of AD from MRI scans.^9–11^ Nowadays, CNN models for image segmentation provide the best performance over alternative image processing strategies and are widely applied in clinical research for brain segmentation into anatomical regions.^12,13^ Commercial MRI volumetry applications, for example icometrix^®^ icobrain dm^®^,^14,15^ Combinostics^®^ cMRI^®^ Dementia Report^®^,^16,17^ NeuroQuant^®^ Dementia,^18^ VUNO Med^®^-DeepBrain^®^,^19^ and Neurophet^®^ AQUA^®^,^20^ use CNN-based brain segmentation pipelines to assist clinicians in discovering disease pathology from brain scans by quantifying brain region volumes compared to normative data. These tools visually summarize volumetric findings in various modes: 1) percentile scores for each region of interest (ROI) in a tabular manner, 2) radar plots, plotting volume percentile scores for the cortex at lobe level granularity, and 3) through subject distribution plots and percentile curves, marking significant atrophy levels for each ROI. However, they only report a small number of pre-defined ROIs based on a priori knowledge and specific diseases relevance, lacking a generalizable framework for aggregating disease pathology at different levels of brain abstractions, e.g., cortical, lobar, hemispherical, etc. Moreover, to date there is only one radiological clinical decision support tool available, Combinostics cNeuro^®^ cDSI,^21^ which supports the differential diagnosis of multiple different types of dementia such as FTLD and AD. cDSI builds upon additive linear models which combine information from various a priori defined neuroanatomical features, cerebrospinal fluid protein measures and neuropsychological assessment data. Extending this basic idea of aggregating clinically relevant information, we aimed at a generalized computational framework that can be readily applied to other neurologic disorders.

Within neuroinformatics, there have been knowledge engineering investigations for creating hierarchical representations of the brain, by delineating conceptual relationships among the anatomical ROIs using formal models, commonly referred to as ‘ontologies’.^22^ The ontology model's hierarchal formalism by design provides the foundation for computational reasoning and inference. However, as far as to authors’ knowledge, ontologies have not been extended to perform computational tasks such as aggregation of pathological information at different anatomical abstractions.

The goal of our study was to extend established volumetric analyses with semantic ontology modeling in order to create a comprehensible disease pattern exploration pipeline. Hence, building upon current tools, we introduce a novel computational framework that integrates the data-driven CNN segmentation models, the computational ontology methods for consolidating pathological information—volumetric and cortical thickness measures, and the visual summary reports to enhance the assessment of atrophy profiles across the entire brain in a single view.

## Methods

**Figure 1.**
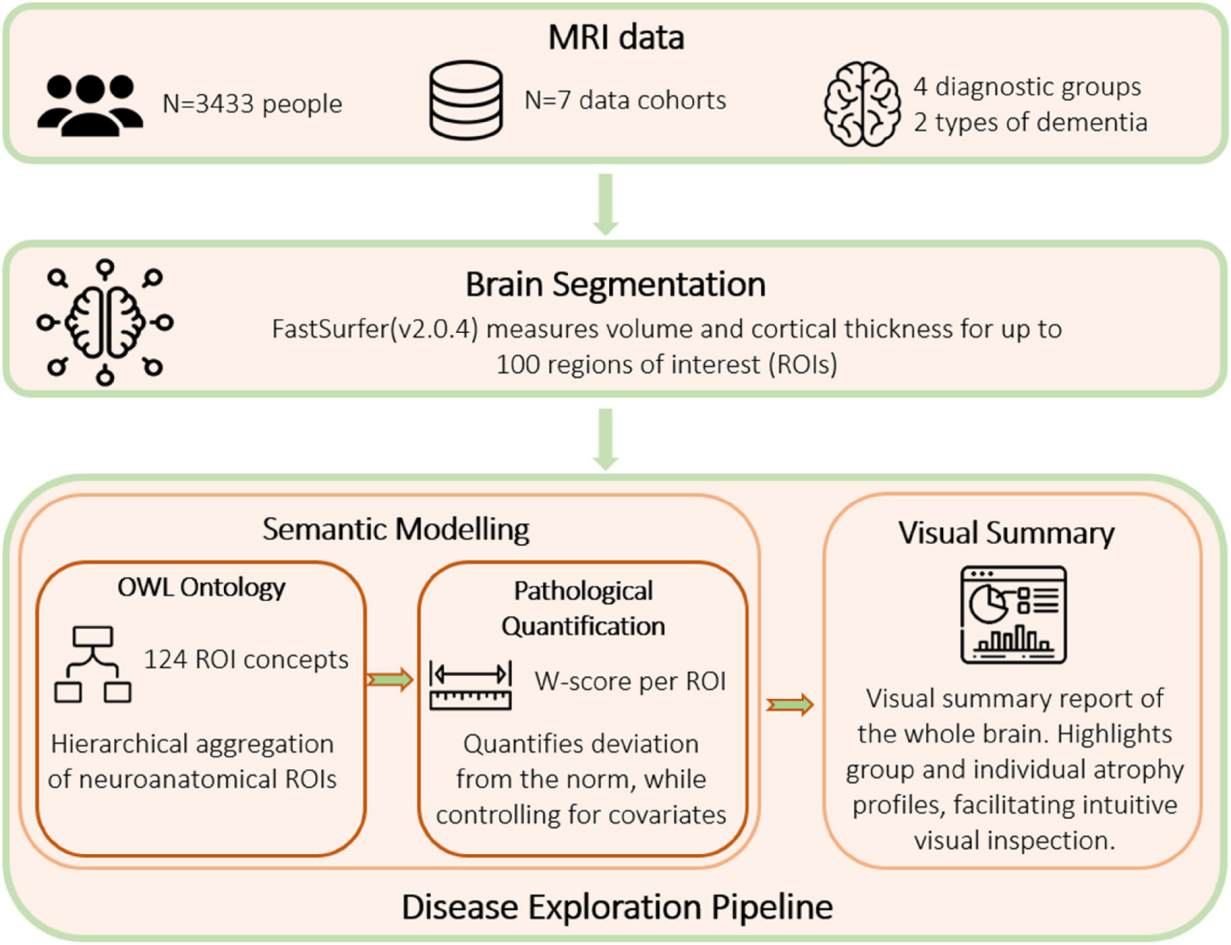
Proposed pipeline for disease exploration. MRI scans were collected from seven study sources, and were preprocessed using the existing brain segmentation tool FastSurfer. Semantic modeling is the first component of our pipeline, where the Web Ontology Language (OWL) model encodes anatomical parent-child relationships, and the pathology quantification step aggregates volume and cortical thickness, at different abstraction levels. Visual summary plots were realized using the sunburst charts, presenting the hierarchy of anatomical relations and volumetric findings.

The workflow for our study is schematically presented in Figure 1, depicting the data set pooled, the preprocessing steps involved, i.e., brain segmentation, and the proposed disease exploration pipeline. Building on our previous short paper, we implemented key enhancements to improve the methodological depth and scientific evaluation of the current study. Specifically, a novel cosine similarity approach was introduced for more precise disease group separation, extending the robustness of visual analysis. The dataset was expanded to include people with bvFTD as additional diagnostic group, and the data modality was extended to include the cortical thickness measures. These broadened the previous focus, which was limited to the volumetric measurements in patients across the AD spectrum. Additionally, an extensive literature review of the ontological studies enriched the theoretical framework, addressing gaps in the earlier conference proceedings publication.^23^

### Neuroimaging datasets and processing

In our study, T1-weighted volumetric MRI scans were obtained from seven study sources: The Alzheimer's Disease Neuroimaging Initiative (ADNI), study phases ADNI2 and ADNI3, the Australian Imaging, Biomarker & Lifestyle Flagship Study of Ageing (AIBL),^24^ the DZNE Longitudinal Study on Cognitive Impairment and Dementia (DELCODE),^25^ the European DTI Study on Dementia (EDSD),^26^ the DZNE Clinical Registry Study on Frontotemporal Dementia (DESCRIBE-FTD), and the Frontotemporal Lobar Degeneration Neuroimaging Initiative (FTLDNI) which is also known as Neuroimaging Initiative in Frontotemporal Dementia (NIFD). Our study included N = 3433 MRI scans in total. In case where scans from multiple time points were available, we considered only the ﬁrst MRI scan from each participant in our study. Table 1 lists the demographic statistics across disease groups. See Supplemental Table 1 for statistics reported for each cohort.

**Table 1.** Sample statistics per disease diagnosis stage and subtype.

Aggregate Statistics / Disease Stages and Subtypes	HC (N = 1625)	MCI (N = 1132)	AD (N = 549)	bvFTD (N = 127)	p
Age	70.4 ± 7.6	72.5 ± 7.4*	74.1 ± 7.7*	62.8 ± 8.2*	<2e-16
MMSE	29.1 ± 1.1	27.6 ± 2*	22.2 ± 4.2*	23.7 ± 5.7*	<2e-16
Education	15.7 ± 3	15.3 ± 3.2*	13.8 ± 3.9*	14.7 ± 3.3*	<2e-16
Sex (F/M)	F: 922 (56.7%) M: 703 (43.3%)	F: 504 (44.5%) M: 628 (55.5%)	F: 280 (51.1%) M: 269 (48.9%)	F: 45 (35.4%) M:82 (64.6%)	1.9e-11

HC: healthy control, MCI: amnestic mild cognitive impairment, AD: dementia due to Alzheimer's disease, bvFTD: behavioral variant frontotemporal dementia, MMSE: mini-mental state examination score, F: female, M: male. Number reported as (mean ± sd). The last column shows the statistical test results from the ANOVA for age, MMSE and education, as well as from the chi-squared test for sex. Asterisks (*) indicate significant differences between groups (p < 0.05) based on pairwise two-sample t-test with HC as reference group.

It should be noted that MCI is a heterogeneous syndrome that could be caused by various underlying conditions. Data cohorts such as ADNI, AIBL and DELCODE, have carefully controlled the subject selection in terms of inclusion/exclusion criteria and have focused primarily on amnestic MCI, i.e., only people with affected memory were included in the MCI group and other conditions such as depression or substance abuse were excluded.

### Anatomical segmentation

We applied the fully automated FastSurfer brain segmentation pipeline as a preprocessing step. The MRI volumes in the native space were bias-field corrected to harmonize image intensities using the N4ITK algorithm, and then directly segmented into anatomical ROIs using FastSurferCNN,^12,13^ a deep convolutional neural network architecture for volumetric segmentation. FastSurferCNN is part of larger FastSurfer tool chain that also performs cortical surface reconstruction and thickness analysis. FastSurferCNN, includes three convolutional neural networks trained in parallel, each limited to one orthogonal 2D brain slice direction (axial, coronal, and sagittal). It uses multi-slice information aggregation, resulting in reduced memory requirements and an overall faster procedure, performing the whole brain segmentation in under a minute. FastSurfer operates in the subjects’ native space and due to its deep learning approach, it allows for the omission of computationally expensive preprocessing steps such as skull stripping and prior registration. FastSurfer follows the Desikan–Killiany–Tourville (DKT) atlas protocol and produces 100 different anatomical segments.^27,28^ After segmentation, the FastSurfer tool chain additionally runs FreeSurfer's *tkregister2* to estimate a linear registration to the Montreal Neurological Institute (MNI) reference space, which is primarily used to derive the total intracranial volume (eTIV) of subjects. We also used the FastSurfer surface reconstruction tool for obtaining cortical thickness estimates.

### Semantic modeling

Following the segmentation of the brain into different anatomical ROIs, we established conceptual relationships among these ROIs in a hierarchical formal model. In accordance with the FMA framework,^22^ we created an ontology in OWL that captures the spatial connections between various brain regions. FastSurfer's 100 DKT-aligned ROIs were structured as leaf nodes in the ontology, aggregated under 24 parent concepts, including the whole brain, hemispheres, and cerebral lobes. The developed ontology model is currently a specific instance tied to the specific brain atlas produced by the FastSurfer segmentation; however, it can be directly adopted to any other kind of neuroanatomical atlas by adjusting the corresponding leaf ROI entities. The computational methodology for the pathology aggregation steps were designed as a generic, ontology-agnostic framework. See Supplemental Material 2 for further details on the development of the anatomical ontology model.

#### Estimation of pathological deviation

Following the brain segmentation, for each ROI we quantified the pathological deviation of the measures—volume and cortical thickness, from healthy control (HC) levels. These deviation metrics were corrected for effects from other covariates. We applied the metric w-score, which is akin to z-scores and quantifies deviation from the expected HC values and is defined as:^29–31^

(1) $$W=\frac{y\;-\;\hat{y}}{SD\;(e_{controls}\;)}$$

where y is the estimated ROI measure obtained from the segmentation tool, SD(.) is the standard deviation and e_controls_ is the residuals of the HC. Here, ŷ, the expected ROI measure, predicted by linear regression model (fixed effect) that controls for the confounding covariates of age, sex, MRI field strength, and brain size (total intracranial volume). For each ROI obtained from the segmentation tool, we trained a linear regression model only on the HC subjects pooled from all data cohorts to provide normative values.

We modeled a hierarchical relationship amongst all ROIs from the segmentation tool which were defined as child nodes, and introduced several parent nodes at higher levels according to anatomical abstractions (e.g., lobes and hemispheres). These hierarchical relationships assisted us in aggregating volumetric and cortical thickness pathology, i.e., the w-score, for both children and parent nodes. Mathematical details for the pathology quantification procedures can be seen in Supplemental Material 3.

The regression models for estimating w-scores were implemented in Python using the scikit-learn package.^32^ The computational ontology framework was developed in Python with the owlready2 package,^33^ which includes an implementation of a SPARQL query engine to provide query/retrieval functionality and logical inference on classes and instances stored in the ontology model.

### Creating visual summary plots

After extracting volumetric deviation scores (w-scores) from the brain, we aimed to present them visually. There was an inherent hierarchical structure of the brain regions. There are various visualization methods for hierarchical structures, such as tree maps: dendrograms and radial trees, network graphs, and sunburst plots. The tree-based visualizations and the network graphs gave higher importance to representing the hierarchical structure, visualized with membership lines, but lack an intuitive visual element for representing other quantities (i.e., the pathological deviations) associated with the hierarchical structure, which are sometimes visualized with node color and size.^34^ We chose the sunburst plots as they represented both the hierarchical structure and the pathological w-scores at an equal footing. We also chose sunburst plots, as they assist in creating a consistent plot appearance across subjects by selecting a common fixed layout.

The sunburst summary charts were realized with Plotly.^35^ They visualized hierarchical concepts within concentric rings, where each ring represented a different level of abstraction. The outermost ring represented the child-ROIs while the successive inner rings represented the parent-ROIs at various levels, and the center represented the root node (i.e., the whole brain). We utilized a customized color scale to indicate the extent of deviation, where a gradual intensification of color signifies increasing pathological observations. Deviations, within the range of −1.5 < w-score < 1.5 units, commonly considered “normal,” were depicted in yellow hues. Conversely, deviations exceeding |w-score| > 2.0 units, indicating significant deviations from normal levels were represented with colors intensifying gradually. Here, red hues indicated atrophy findings, characterized by negative w-scores, while green hues signified enlargement findings, associated with positive w-scores. Beyond the summary plots, we also devised 3D overlay plots in which the ROI segmentations were highlighted in the same color scale as described before. Here, the associated w-score guide the color intensity, demarking pathological ROI findings.

There are several trade-offs that need to be considered when visualizing multiple sources of information, i.e., including both volumetry or cortical thickness in the presentation of summary plots. These comprise i) the application of so-called bi-variate color palettes to highlight concordance or discordance between the metrics, also known as ‘(visual) feature fusion’, ii) the derivation of a composite metric (e.g., a ratio, min or max) for a uni-dimensional representation, also known as ‘data fusion’, or iii) the use of alternative visual encoding channels such as style or texture.^36,37^ Even though these visualization trade-offs are non-trivial, we chose to present the summary plots using the maximum operator over each region, as an initial solution for merging the information sources.

### Similarity quantification of atrophy profiles

We implemented a mechanism for computing the similarity between w-score profiles, e.g., for comparing group-averages or single-subject data for different stages of AD and types of dementia. We chose the cosine similarity as the similarity metric. It ranges between [^−1,1^], where a similarity measure of 1 (or −1) marks identical (or semantically opposite) w-score profiles, and a measure of 0 marks unrelated w-score profiles. Notably, the cosine similarity quantified the angle between the multidimensional vector representation of the region-specific w-scores, irrespective of the actual magnitude of w-scores. Thus, it was more sensitive to the patterns of simultaneously deviating ROIs than alternative similarity metrics such as Euclidean distance. The cosine similarity was calculated across all the ROIs (including parent and child ROIs). The similarity measurement between a subject and the disease stages’ mean w-score profiles quantified the likeness to the closest disease stage, and provided statistical evidence beyond the subjective visual comparison.

Specifically, for the group separation task between dementia types, we wanted to quantify if the cosine similarity metric can reliably distinguish between the two: AD and bvFTD. It was of interest for us to understand the amount of group information captured by the w-score profile's cosine similarity. Thus, we established a metric ‘Disease Similarity Index (DSimI)’, defined as:

(2) $$DSimI(x)=\cos\;(x,AD_{w-score})-\cos\;(x,\;bvFTD_{w-score})$$

where x is a subject's w-score profile, i.e., the vector of w-scores for each ROI, cos(.) is the cosine similarity metric, and AD_w−score_ or bvFTD_w−score_ are the mean w-score profiles for the AD or bvFTD groups, respectively. We simultaneously utilized both, the volumetric and the cortical thickness w-scores.

## Results

### Semantic modeling

**Figure 2.**
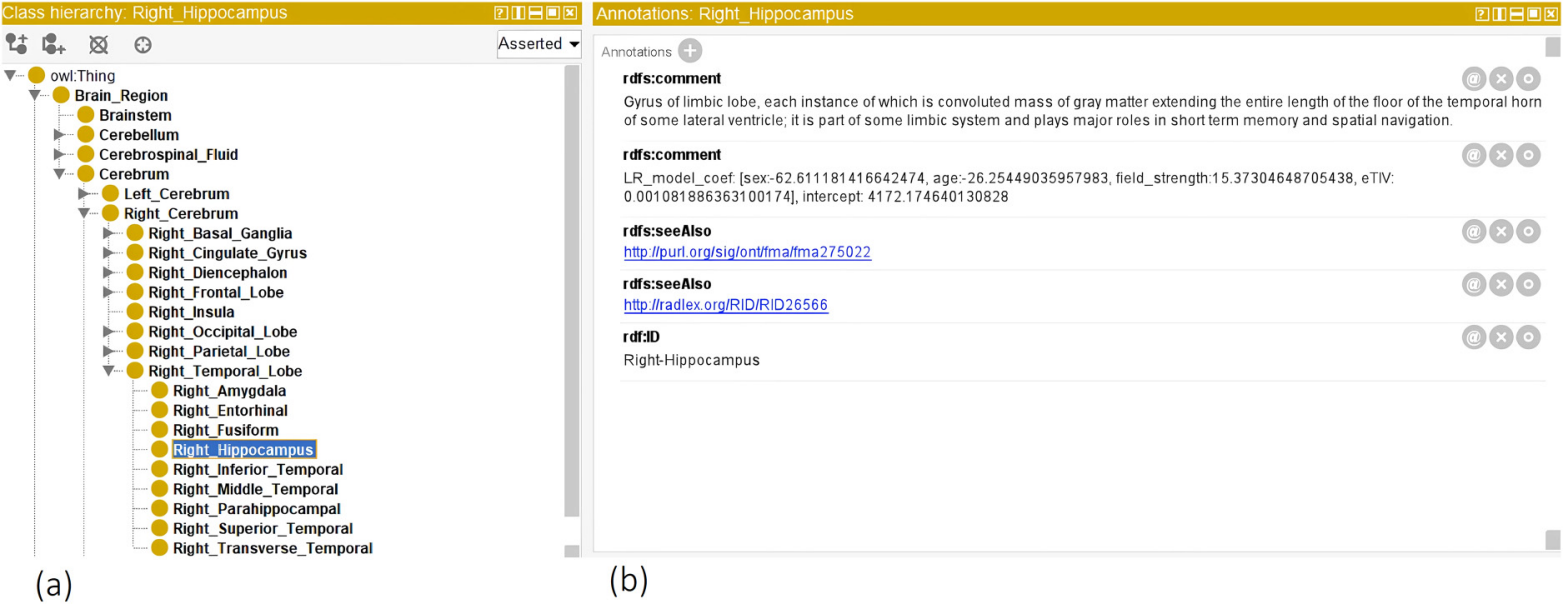
Illustrates the OWL ontology, which encodes semantic a priori information about brain structure. (a) The leaf nodes (or classes) are the ROI concepts segmented by the FastSurfer tool. Parent nodes were derived from a priori neuroanatomical knowledge. (b) The annotation window illustrates ROI properties, for example for the right Hippocampus region, there are (i) as ‘comment’ the textual definitions, (ii) ‘seeAlso’ tags linking to FMA and RadLex ontologies, (iii) linear regression model coefficients, and (iv) the ROI ‘ID’ for FastSurfer mapping. Note that model coefficients are raw values and not standardized beta values.

For encoding the a priori neuroanatomical structure, we developed an OWL ontology, illustrated in Figure 2. The source code files and the ontology were made available via GitHub and BioPortal (please see the section ‘Data availability’).

**Figure 3.**
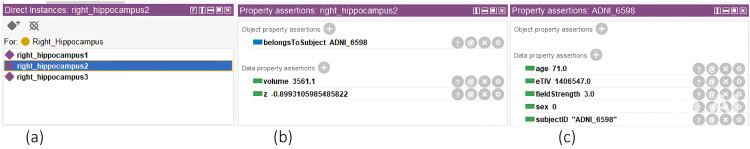
The class instances windows, illustrating the participant's right hippocampus volumetric levels. In (a), we see the database of all instances of the ‘right hippocampus’ class. In (b), we see the volume (in mm^3^) and the volumetric deviation (‘z’ as w-score). In (c), we see the covariates for an individual.

As outlined in the methods section, we trained linear regression models for the child-ROIs and parent-ROIs, predicting expected measurements controlling for covariates—age, sex, head size (eTIV) and MRI scanner magnetic field strength. We stored model parameters in the OWL ontology as annotations of the ROI classes. In Figure 2(b), we illustrate the trained linear regression model coefficients for a single ROI, e.g., the right hippocampus. After initializing the linear regression model at all ROI levels, we calculated the w-scores for all the subjects. These w-scores and the raw measures were stored in the ontology under the ROI instance objects as data properties. Figure 3 illustrates one such example.

### Visual summary plots of pathology

#### Application to single-subject data

**Figure 4.**
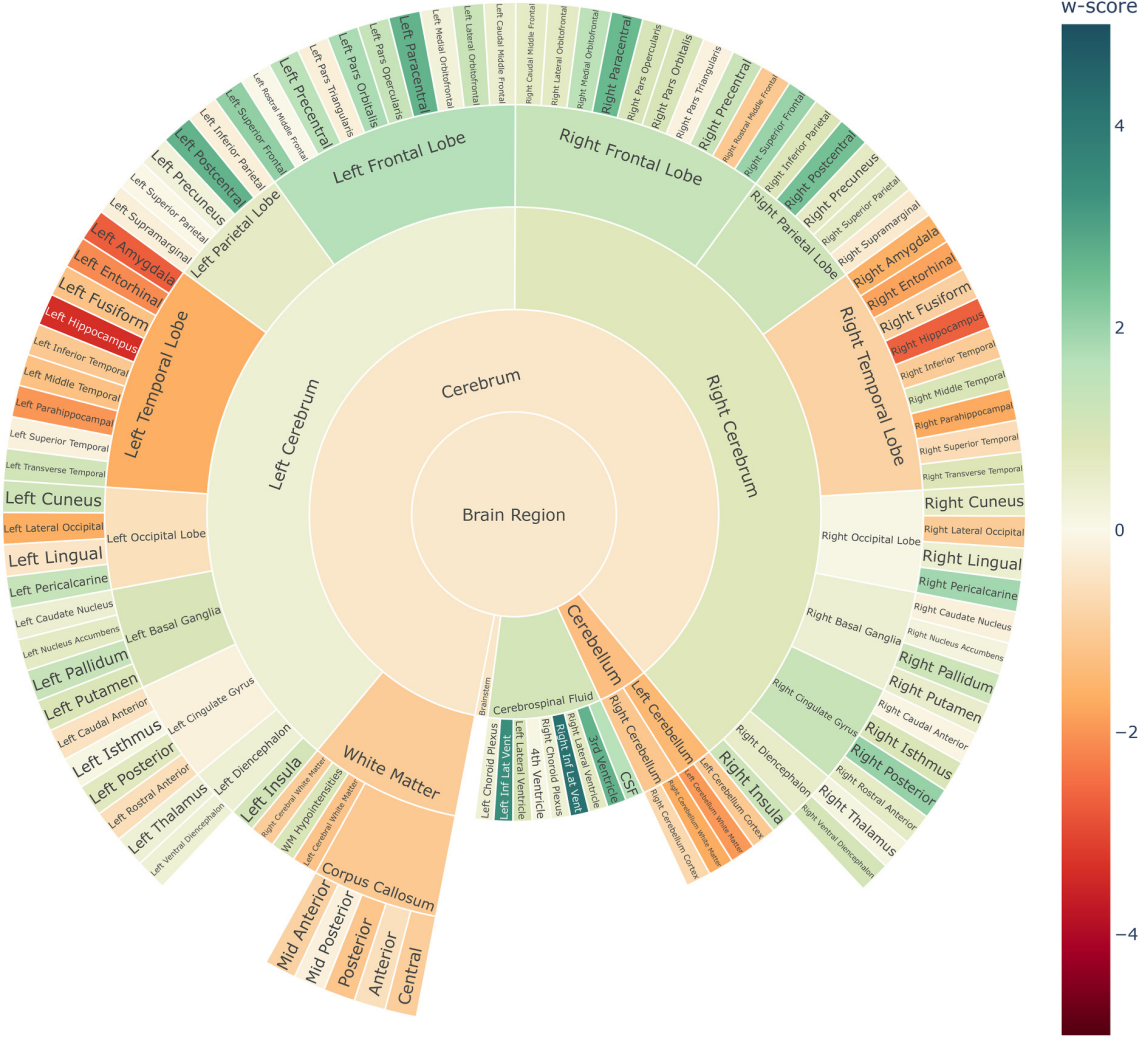
Single-subject volume sunburst chart for the ADNI Alzheimer's patient ID 6849 with mild dementia.

**Figure 5.**
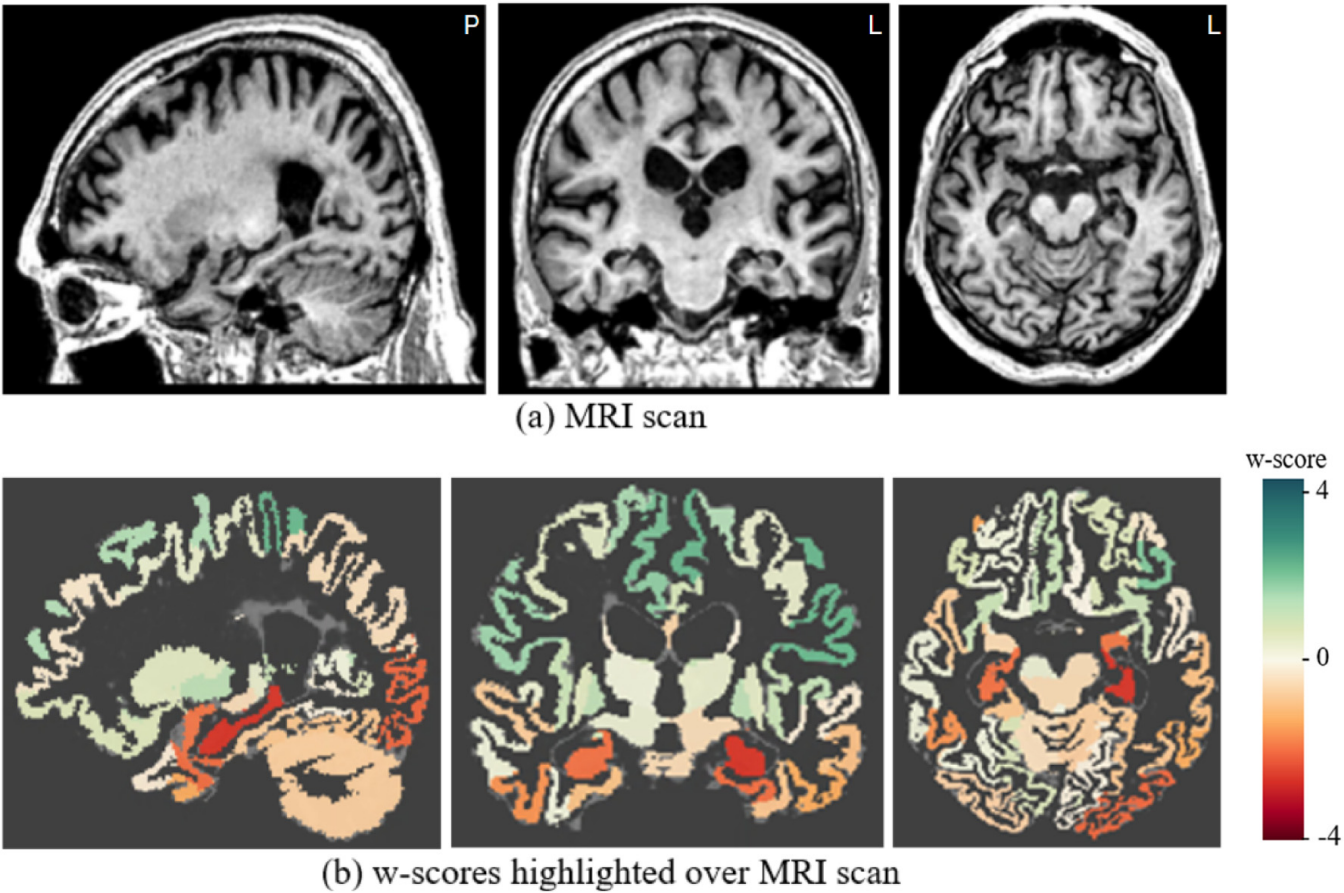
Single-subject's (a) MRI scan and (b) overlaid volumetric w-score map for the ADNI Alzheimer’s patient ID 6849 with mild dementia.

Figure 4 illustrates sunburst chart and MRI scans for one arbitrarily selected individual from the ADNI data cohort, suffering from dementia due to AD. The subject ID 6849 from the ADNI3 data cohort, is a 78-year-old male with 16 years of education and a MMSE score of 21, indicating mild dementia. This patient showed local atrophy patterns, with volumetric loss located around the temporal lobes (Figures 4 and 5). Supplemental Figure 2 illustrates the average cortical thickness sunburst chart for the same subject, while the Supplemental Figure 3 illustrates the region-wise maximum pathology sunburst chart.

The cosine similarity between the subject's w-score profile and the group average w-score profiles were: −0.23 for HC, 0.47 for AD and 0.29 for bvFTD, respectively, which yields a positive DSimI of 0.18, with AD being the most similar w-score profile. The patient showed slightly asymmetric levels of atrophy between the hemispheres. The hippocampus and the temporal lobe ROIs showed the most pronounced atrophy, indicated by the highest w-scores.

#### Visualizing the trajectory of disease severity on group-level

**Figure 6.**
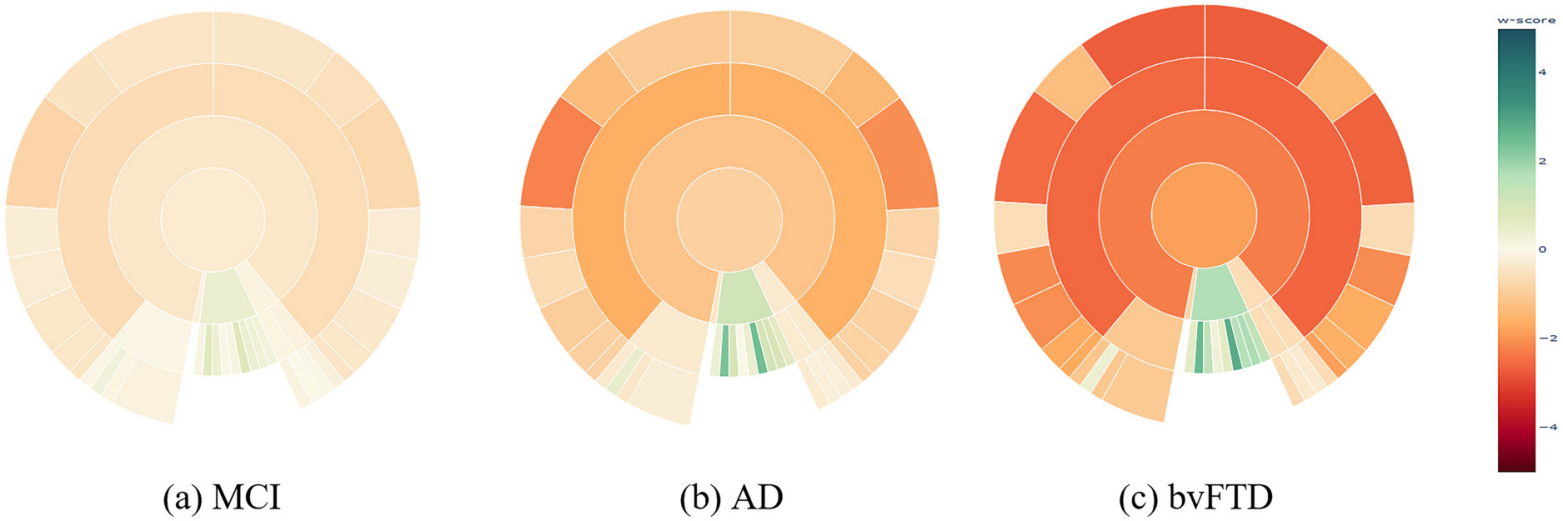
Sunburst summary plots illustrating mean volume w-scores across ROIs at the lobe level, highlighting differences between disease stages of AD, and dementia types. MCI: mild cognitive impairment; AD: dementia due to Alzheimer's disease; bvFTD: behavioral variant of frontotemporal dementia. Please refer to the online version of the plots for a more detailed visualization.

The summary plots depicted in Figure 6(a) and 6(b) demonstrate a gradual decline in the w-scores of the temporal lobe, corresponding to the increasing severity of (volumetric) atrophy findings typically associated with the progression of AD (statistically significant in one way ANOVA with F-values of 312.4 and 385.1, df = 2, p < 0.001, for the right and left temporal lobes, respectively). See Supplemental Figures 4–7 for more extensive illustrations of the volumetric summary plots across different clinical stages and data cohorts, while the Supplemental Figure 8 illustrate the mean cortical thickness summary plots, respectively, across different clinical stages and types.

**Figure 7.**
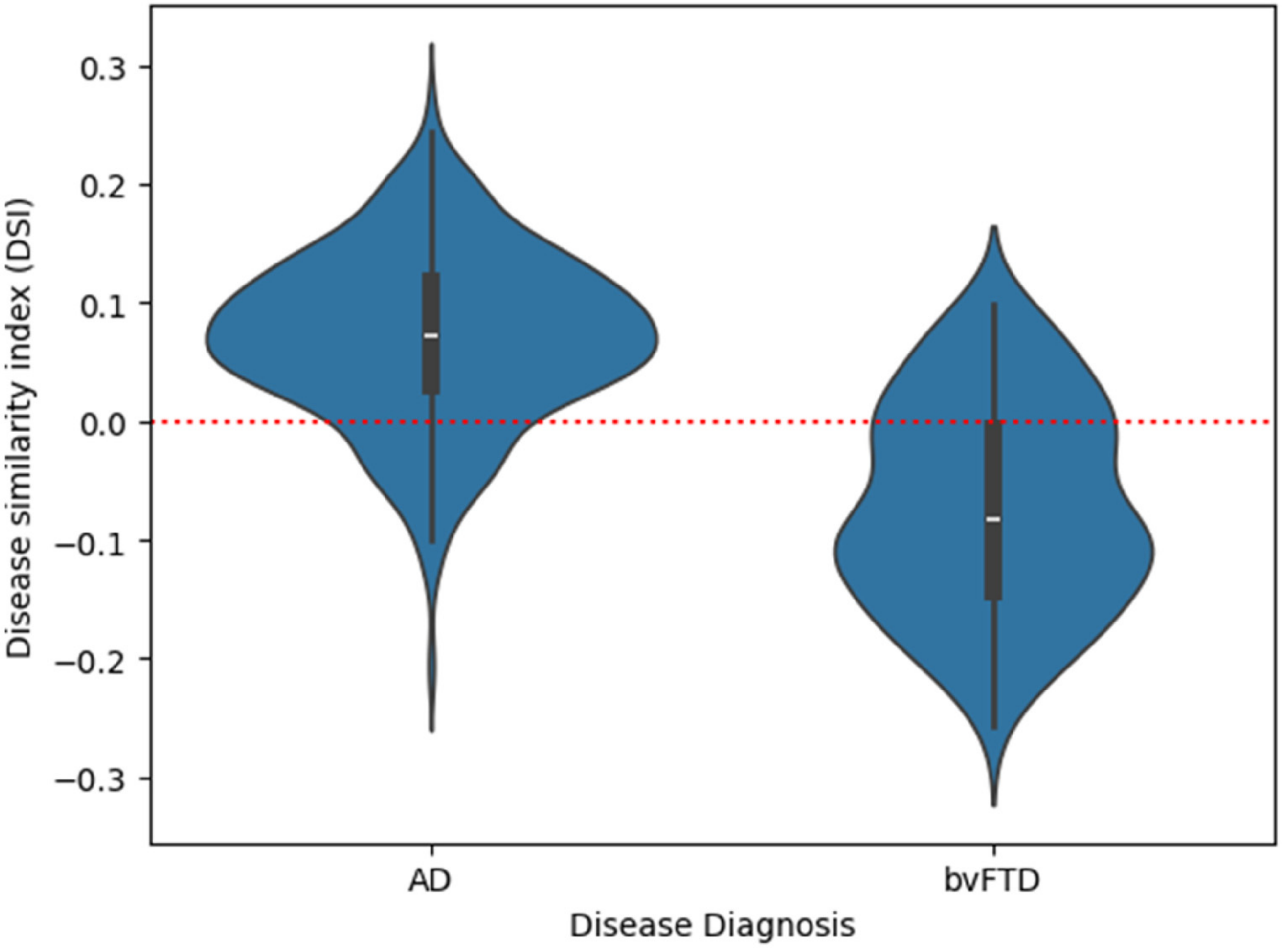
The Disease Similarity Index (DSimI) for the diseased subjects (AD or bvFTD). A red horizontal line marks the threshold at 0 (i.e., decision boundary) for separating the two diseased types.

#### Comparing neurodegenerative diseases

Figure 6(b) and 6(c) illustrates the summary plot showing differential patterns of atrophy among the two dementia types. Figure 6(b) shows dementia due to AD versus bvFTD (Figure 6(c)). The frontal lobe's volumetric w-scores differed statistically highly significant between both dementia types for both the right and left frontal lobes (Welch's t-test, left frontal lobe: t = 8.94, df = 147.0, p < 0.001; right frontal lobe: t = 9.17, df = 150.1, p < 0.001). The effect size was large, with a Cohen's d = 1.2 for both. This comparison of w-scores suggests that the visual impression reflects a true difference in the mean w-score profiles between the dementia types.

#### Cosine similarity metric for dementia type separation

Although the cosine similarity between the mean w-scores profiles of AD and bvFTD was found to be high with 0.89, it contained useful information about the most likely group. Figure 7 illustrates a violin plot of the Disease Similarity Index (DSimI) for the people with dementia (AD or bvFTD). We see a separation of the two diseased types - AD and bvFTD, and using the DSimI as classification score thresholded at the zero point yielded an area under the curve (AUC) metric of 0.89 in receiver operating characteristic (ROC) analysis. In an extended 10-fold cross validation analysis, the ROC-AUC for the whole brain was 0.88 ± 0.05. The evaluation metrics from 10-fold cross validation were - balanced accuracy (bacc): 0.80 ± 0.04, sensitivity: 0.75 ± 0.06, specificity: 0.84 ± 0.04, and the F1-score: 0.65 ± 0.07. Table 2 reports the classification scores yielded by considering different regional subsets. See Supplemental Material 8 for a more extensive discussion of the DSimI.

**Table 2.** Incremental effects of considering different regions on the Disease Similarity Index (DSimI) in a 10-Fold Cross Validation.

Regions Considered in DSimI	AUC	Balanced accuracy
Amygdala	0.53 ± 0.08	0.51 ± 0.06
Hippocampus	0.60 ± 0.06	0.56 ± 0.06
Temporal Lobe	0.72 ± 0.13	0.69 ± 0.08
Frontal Lobe	0.67 ± 0.06	0.64 ± 0.06
Frontal and Temporal Lobe	0.83 ± 0.06	0.78 ± 0.06
Cerebrum	0.88 ± 0.05	0.78 ± 0.05
Whole Brain	**0.88 **± **0.05**	**0.80 **± **0.04**

## Discussion

In this research, we introduced a methodological framework for assessing and presenting MRI scan-based brain atrophy measures. Each component of our two-part pipeline was built modularly and can be altered according to the needs of the clinicians, with the possibility of modifying the leaf nodes corresponding to a specific brain atlas, the hierarchical arrangement of the ROIs within the ontology, and the appearance of summary plots. We also implemented a computational approach for quantifying the subject-to-group-mean similarity of the w-score profiles. Creating our own ontology was essential because existing ones either did not provide an explicit hierarchical structure for the relations between ROIs,^4,38–43^ or lacked computational reasoning,^22,44^ hindering the aggregation of pathological data across various levels of abstraction. Similarly, we aimed to develop a flexible generic visualization framework that extends current MRI volumetry software,^14–20^ which address only very specific use cases and cannot be directly applied to other diseases.

The unique aspect of our disease exploration pipeline is the extension of the neuroanatomy ontology with the ability for computational reasoning, for instance integrating an efficient method for quantifying pathological information, and generation of the summary visualizations. Notably, the quantification of volumetric deviations^29–31^ provides a prototypical proof-of-concept of the computational capabilities of such an ontology framework, to which we aim to add similar aggregation mechanisms for other pathological information such as cortical thickness measures, tracer uptake values of positron emission tomography (PET) studies, or relevance scores provided by deep learning methods.^45^ The additive linear regression (LR) models could efficiently utilize the parent-child relationships among brain regions for recursive inference. The summary plots are interactive visual representations and illustrate distinct atrophy profiles associated with different disease types and stages. The proof-of-concept results obtained from the subject-to-group-means similarity quantification and using this for estimating the most likely dementia type are promising. This explorative reporting of pathological brain atrophy findings could likely assist clinicians in discriminating between different pathologies in an interpretable and reliable manner.

Our semantic modeling component, i.e., the OWL ontology, descriptively models the well-established neuroanatomical hierarchy of abstraction levels and regions. Based on this explicit encoding of structural information and parent-children associations, we can now develop more extensive semantic knowledge networks. These knowledge networks open up space for creating clinical decision support systems that directly integrate recommendations from diagnostic guidelines within established data driven analyses.^5^

We optimized our summary plots to reflect the symmetry of the brain hemispheres and to list the lobes in anatomical order. The interactive nature of the Plotly library enables one to control the amount of information illustrated in the sunburst summary plots. This enables developers to choose if they wish to show only the aggregated volumetric w-scores at the lobe abstraction level, initially hiding the outer ring of individual brain regions, which then can be displayed by users on demand by clicking on the respective parent entity. In the presented figures, the sunburst plots offer a quick and comprehensive overview of volumetric w-scores across all brain regions simultaneously. They provide an easy-to-understand visualization of both gray matter atrophy and ventricular enlargement with just a single glance. This is a feature novel to our summary plots compared to previous MRI volumetry reporting modalities, which is primarily based on displaying aggregated information in large tables.^19^ Although we included inputs from physicians and visualization experts in the development of the summary plot design, further exploration and improvement of the usability and utility is beyond the scope of the current study and will be addressed in future work.

From Figure 6(a) and 6(b), we see the gradual spreading of atrophy across different disease stages. By definition we see no atrophy, i.e., w-score = 0 for HC, followed by selective medial temporal lobe atrophy in amnestic MCI, and widespread cortical atrophy in dementia due to AD, which is coherent with the literature regarding AD progression.^46^ There is also an expansion of the inferior lateral ventricles. These findings visualized in the sunburst charts are in alignment with the expected volumetric pathology patterns found in AD.^47^ From summary plots, Figure 6(b) and 6(c), we also see distinctive patterns for the two dementia types. AD patients show atrophy in the temporal lobe and enlargement of the inferior lateral ventricles (Figure 6(b)), in red and green hues respectively. Patients with the bvFTD additionally show atrophy patterns in the frontal lobe (Figure 6(c)), as expected.^48,49^ It is interesting to see relatively more intense atrophy levels (w-scores) in the cerebrum in bvFTD than in the AD subjects. This difference in the w-scores might be partly explained by the differences in age ranges between the diagnoses, with bvFTD being more frequent among relatively younger patients (mean age of 62.8 years) versus AD being more frequent in older patients (mean age of 74.1 years), see Table 1. Thus, our linear regression models, controlling for aging effects, could have slightly overestimated the volume deviations in the younger bvFTD subjects due to the fact that 1) the majority of the normative cognitively normal population used for training was from relatively older people (mean age 70.4 years), i.e., most HC subjects were closer in chronological age to AD patients than to the bvFTD patients, and 2) the underlying implicit assumption of linear regression models of a linear association between age and volumes,^50,51^ which might be addressed by more complex modeling strategies such as spline or Gaussian process regression in future work.

Comparing Figure 4 and Figure 6 highlights the potential capability of using the single-subject w-score profiles in mapping individuals to a most probable dementia type based on similarity quantification. Therefore, we quantified the subject-to-group average similarity using the cosine similarity metric. Quantifying w-score similarity produces a metric that would be akin to already existing visual rating scales, such as the global cortical atrophy scale and medial temporal atrophy scale being widely used by radiologists.^5^ The prototypical similarity mechanism provides objective evidence beyond the subjective judgments of radiologists. Using the similarity metric for distinguishing disease type, we obtained a balanced accuracy of 80 ± 4% and AUC of 0.88 ± 0.05 for separating the AD and bvFTD groups. These performance measures are comparable with previous studies that explored various modeling strategies such as frontal-temporal volumetric ratios, or imaging markers derived from disease-sensitive regions.^52–55^ Some studies suggested a multimodal approach by incorporating information sources beyond MRI scans.^21,56^ The cDSI study reported a comparable balanced accuracy of 80.3%.^21^ Two other studies evaluated the radiologist's accuracy levels for the AD-vs-FTD separation task based on MRI scans and reported accuracy values between 68.6% and 73%.^57,58^ Click or tap here to enter text. Though one should be mindful while interpreting the performance measures from the expert rater studies, as they often include only a limited number of expert raters and scans evaluated.

While considering different anatomical subsets while evaluating the DSimI AUC for separating the AD and bvFTD subjects (see Table 2), we see the incremental effects of considering atrophy signals, i.e., w-score similarity, from more anatomical regions. Interestingly, an acceptable differential diagnosis could be made by just capturing the signals from temporal and frontal lobes together (mean AUC: 0.83 ± 0.06). It should be noted that these DSimI metrics consider both volumetric atrophy and cortical thickness measures. We found that adding cortical thickness measures to the DSimI generally improves the AUC metrics by 2 to 5 percentage points, suggesting a complimentary nature of the pathology ‘signature’ captured by the two measures, which was also found by one previous study.^59^

The similarity metrics and summary maps together could provide a nuanced, automated, regionally specific, and visually intuitive way of measuring atrophy profiles, which could be as useful as the traditional visual rating scales. The cosine metric is an established similarity metric used in various applications, such as recommender systems^60^ and text analysis.^61^ Further benchmarking various alternative modeling strategies for the separation of AD-vs-bvFTD, for example, by more sophisticated feature selection, is beyond the scope of the current study. Notably, the high variation in single-subject w-scores requires careful interpretation by the radiologists and necessitates referring back to the MRI scan and the representative group mean summary plots of the different disease stages and types.

### Limitations and future outlook

Various deep learning based whole-brain segmentation tools have been proposed over the years.^62–66^ These models often make advancements to the underlying CNN architectures used for information processing and at times have different ROI parcellation paradigms. However, analyses performed by multiple studies have shown the relative stability of FastSurfer.^12,64,66^ Hence, a different choice of segmentation tool or neuroanatomical atlas could alter the list of children nodes (ROIs) introduced in our study, but the parent aggregations should remain comparatively stable against these choices, as this is the benefit of aggregating information at higher abstraction levels. Thus, the specific choice of segmentation tool is expected to not alter the overarching findings of our study.

We intend to perform testing of the diagnostic utility of the presented framework with clinical experts, to further evaluate our pipeline and collect feedback for improvements. Future work also includes accounting for longitudinal data or multiple imaging sessions in the ontology. Integration of more sophisticated statistical modeling for controlling volumetric deviation quantification could be achieved with mixed-effect models or other data harmonization approaches such as ComBat.^67^ However, inclusion of non-linear modeling techniques is expected to be of limited utility for now, as i) this would require a much larger normative cohort of HC subjects^68^ and ii) linear models often have been shown to provide reasonable accuracy within the desired age range of 60 years and older.^69,70^

Another evident addition to this study would be the inclusion of more data cohorts, particularly with FTD subjects (including other FTD phenotypes - semantic PPA and non-fluent PPA), subjects from other neurodegenerative diseases, or specific phenotypes of AD like the logopenic PPA or posterior cortical atrophy. In future, it would be of value, to clearly identify underlying disease copathologies and study their different pathologic signatures based on postmortem data. While calculating the deviation metric with w-scores, we also plan to include other anatomical and functional ROI measurements, such as anatomical shape, or PET tracer uptake.

### Conclusions

This study introduces a two-part framework for accurately quantifying and reporting volume-based pathology found in brain MRI scans. The components consist of a semantic ontology model and a computational framework that generates hierarchical summary plots. Additionally, a prototypical method for assessing similarity between w-scores profiles was presented. We situated and adapted both components in the larger context of dementia detection, but they were designed in a modular fashion for high flexibility. The semantic ontology incorporates neuroanatomical relationships, explicitly encoding a priori structural information about brain regions, and allows for efficient volumetric deviation calculation. The summary plots offer intuitive visualizations of volumetric deviations across different levels of brain regions simultaneously, capable of creating representations either for individual subjects or group aggregates. Overall, this pipeline offers an automated and efficient method for reporting pathological brain atrophy findings.

## Supplemental Material

sj-docx-1-alz-10.1177_13872877251331222 - Supplemental material for A computational ontology framework for the synthesis of multi-level pathology reports from brain MRI scans Supplemental material, sj-docx-1-alz-10.1177_13872877251331222 for A computational ontology framework for the synthesis of multi-level pathology reports from brain MRI scans by Devesh Singh, Alice Grazia, Achim Reiz, Andreas Hermann, Slawek Altenstein, Lukas Beichert, Alexander Bernhardt, Katharina Buerger, Michaela Butryn, Peter Dechent, Emrah Duezel, Michael Ewers, Klaus Fliessbach, Silka D Freiesleben, Wenzel Glanz, Stefan Hetzer, Daniel Janowitz, Ingo Kilimann, Okka Kimmich, Christoph Laske, Johannes Levin, Andrea Lohse, Falk Luesebrink, Matthias Munk, Robert Perneczky, Oliver Peters, Lukas Preis, Josef Priller, Johannes Prudlo, Boris S Rauchmann, Ayda Rostamzadeh, Nina Roy-Kluth, Klaus Scheffler, Anja Schneider, Luisa S Schneider, Björn H Schott, Annika Spottke, Eike J Spruth, Matthis Synofzik, Jens Wiltfang, Frank Jessen, Stefan J Teipel, Martin Dyrba, , , and in Journal of Alzheimer's Disease
