# Supplementary material for: A computational ontology framework for the synthesis of multi-level pathology reports from brain MRI scans
Source: J Alzheimers Dis. 2025 Apr 21;108(1 Suppl):S258–73. doi: 10.1177/13872877251331222 (PMC12583655; doi:10.1177/13872877251331222)

**Supplemental Material**

**A computational ontology framework for the synthesis of multi-level pathology reports from brain MRI scans**

**Supplemental Material 1. Neuroimaging datasets**

**Supplemental Table 1.** Sample statistics per disease diagnosis stage and subtype.

| **Data Cohort** | **HC** | **MCI** | **AD** | **bvFTD** | **p** |
| --- | --- | --- | --- | --- | --- |
| **ADNI2 (#Subjects)** | **253** | **526** | **187** |  |  |
| Age (SD) | 75.4 ± 6.6 | 72.6 ± 7.8 | 75 ± 8 |  | 9.39e-07 *** |
| MMSE (SD) | 29.1 ± 1.2 | 28 ± 1.7 | 22.6 ± 3.2 |  | <2e-16 *** |
| Education (SD) | 16.6 ± 2.7 | 16.1 ± 2.7 | 15.9 ± 2.7 |  | 0.165 |
| Sex (F/M) | 129 / 124 | 225 / 301 | 78 / 109 |  | 0.06361 |
| **ADNI3 (#Subjects)** | **325** | **185** | **62** |  |  |
| Age (SD) | 70.2 ± 6.4 | 72.2 ± 7.5 | 74.8 ± 7.7 |  | 2.08e-06 *** |
| MMSE (SD) | 29.1 ± 1.1 | 27.8 ± 2 | 23.1 ± 3.3 |  | <2e-16 *** |
| Education (SD) | 16.6 ± 2.2 | 16.6 ± 2.5 | 16.5 ± 2.4 |  | 0.921 |
| Sex (F/M) | 210 / 115 | 84 / 101 | 27 / 35 |  | 1.56e-05 |
| **AIBL (#Subjects)** | **448** | **96** | **62** |  |  |
| Age (SD) | 72.4 ± 6.2 | 74.3 ± 6.9 | 73.2 ± 7.3 |  | 0.0271 * |
| MMSE (SD) | 28.7 ± 1.2 | 27 ± 2.2 | 21.2 ± 5.3 |  | <2e-16 *** |
| Sex (F/M) | 260 / 188 | 47 / 49 | 36 / 26 |  | 0.2643 |
| **DELCODE (#Subjects)** | **214** | **155** | **104** |  |  |
| Age (SD) | 69 ± 5.5 | 72.5 ± 5.7 | 74.6 ± 6.2 |  | 2.86e-16 *** |
| MMSE (SD) | 29.5 ± 0.8 | 27.8 ± 2 | 23.1 ± 3.2 |  | <2e-16 *** |
| Education (SD) | 14.7 ± 2.7 | 14 ± 3.1 | 12.9 ± 3.1 |  | 2.32e-06 *** |
| Sex (F/M) | 124 / 90 | 72 / 83 | 61 / 43 |  | 0.04721 |
| **DESCRIBE (#Subjects)** | **58** |  |  | **61** |  |
| Age (SD) | 60.6 ± 13.4 |  |  | 64.1 ± 9.5 | 0.099 |
| MMSE (SD) | 29.1 ± 1.2 |  |  | 23 ± 6.9 | 1.53e-09 *** |
| Education (SD) | 15.4 ± 2.6 |  |  | 13.5 ± 3.2 | 0.000544 *** |
| Sex (F/M) | 29 / 29 |  |  | 24 / 37 | 0.3796 |
| **EDSD (#Subjects)** | **194** | **170** | **134** |  |  |
| Age (SD) | 68.6 ± 6 | 71.1 ± 7.4 | 72.7 ± 8.3 |  | 2.33e-06 *** |
| MMSE (SD) | 28.9 ± 1.1 | 26.5 ± 2.2 | 20.9 ± 5.3 |  | <2e-16 *** |
| Education (SD) | 13.1 ± 3.7 | 12.3 ± 3.3 | 10.2 ± 3.4 |  | 1.28e-11 *** |
| Sex (F/M) | 97 / 97 | 76 / 94 | 78 / 56 |  | 0.07263 |
| **NIFD (#Subjects)** | **133** |  |  | **66** |  |
| Age (SD) | 63.3 ± 7.3 |  |  | 61.6 ± 6.6 | 0.1 |
| MMSE (SD) | 29.4 ± 0.8 |  |  | 24.3 ± 4.4 | <2e-16 *** |
| Education (SD) | 17.3 ± 1.9 |  |  | 15.8 ± 2.9 | 2.16e-05 *** |
| Sex (F/M) | 74 / 59 |  |  | 21 / 45 | 0.003342 |

For the AIBL dataset, information about years of education was not available. HC: healthy control, MCI: mild cognitive impairment, AD: dementia due to Alzheimer’s disease, bvFTD: behavioral variant frontotemporal dementia, MMSE: mini-mental state examination score, F: female, M: male, SD: standard deviation. Within each cohort, we performed the ANOVA test across groups for the variables age, MMSE and education, and the chi-squared test for the sex variable. Significance codes represent, ***p<0.001; **p<0.01; *p<0.05, uncorrected for multiple comparisons.

**Supplemental Material 2. Development of the anatomical ontology**

Within the medical bioinformatics, there were various projects modeling human anatomy through ontologies. Supplemental Table 2 provides an overview of ontological studies that represented neuroanatomy with different conceptual abstractions and relationships among them. The Foundational Model of Anatomy (FMA) (Rosse and Mejino, 2003), a knowledge-driven framework aimed at harmonizing ontologies across different application domains with respect to anatomy models. However, previous ontologies (Nichols et al., 2014) and various ontology guideline frameworks (Boeker et al., 2011; Dumontier et al., 2014; Jackson et al., 2021) did not yet provide a suitable structure for performing computational reasoning over its class concepts. For example, some semantic concepts were fragmented into functional and anatomical components, but lacked links to each other.

Thus, combining a review of existing ontologies (see Supplemental Table 2) and expert interviews with neurologists and radiologists (N=4), we developed our own formal neuroanatomical ontology model. The expert interviews guided the formation and revision of a regional hierarchy applicable for volumetric analyses as well as visualization and layout aspects to better match neuroradiological reporting conventions. Supplemental Figure 1 illustrates the process of semi-structured expert interviews.


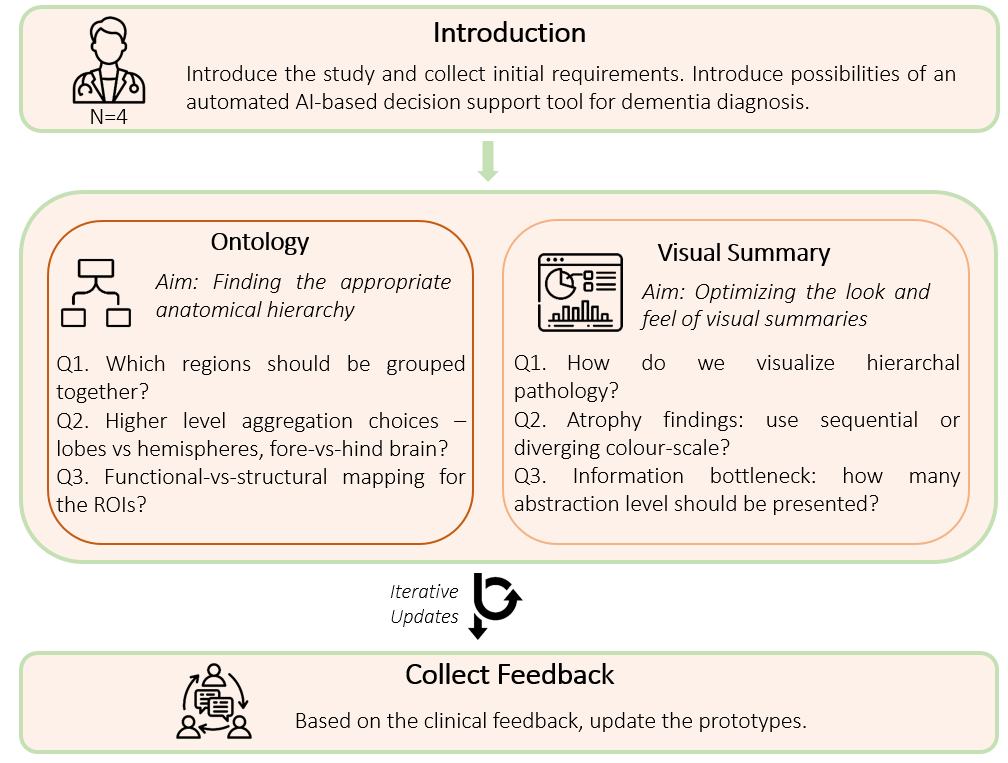


**Supplemental Figure 1.** Semi-structured expert interview flowchart. We collected the clinical feedback on different aspects such as anatomical coherence and visual representation, and integrated these suggestions within the ontology model, inference and visualization tool chain. The interviews approximately lasted for half an hour. These interviews served as a basis for making the overall tool chain more suitable for the volumetry analyses of MRI scans for dementia diagnosis.

In accordance with the FMA framework, we created an ontology in the Web Ontology Language (OWL) that captures the spatial connections between various brain regions. Our model follows selected guidelines for ontology modeling and also adheres to the recommendation of reporting ontology metadata and descriptions of the concepts (Jackson et al., 2021). As the segmentation tool FastSurfer produced 100 ROIs aligned with the DKT atlas, our ontology model initialized these ROIs as leaf nodes of a tree structure, and then aggregated them under different concepts at multiple abstraction levels, which formed the parent nodes of the tree structure. More specifically, in top down order the parent nodes were as follows - the whole brain, cerebrum and cerebellum, left and right hemispheres, and cerebral lobes within the hemispheres. There were 24 such parent concepts that were added. To encode parent-child relations amongst ROIs, we modeled child-ROIs as subclasses of parent-ROIs and also associated an inverse property called ‘hasRegions’ with the parent-ROI. This is logically equivalent to the ‘part-of’ relations reported by other ontology studies listed in Table 1. We created the ‘Subject’ OWL class to reflect the logical concept of an individual patient (data-sample). Instances of this class store metadata and covariate information, that were encoded as OWL data properties. The subject instances were named using cohort and participant identifiers, e.g., ‘ADNI_6598’. We also created a ‘belongsToSubject’ object property to reflect many-to-one relational mapping between the ROI instances and the subject instance. To all ROIs, we added textual definitions based on the neuroanatomical lexicon, and other web resources. When ROIs were found in the other ontologies FMA or RadLex which reflect the identical semantic concept, we added a link to the respective ROI ID (Rosse and Mejino, 2003; Mejino et al., 2008). Notably, of the total number of class concepts present in our ontology model, 79% of these could be found in FMA ontology model and 76% could be found in RadLex ontology model (Mejino et al., 2008).

**Supplemental Table 2.** Comparing proposed ontology model with existing neuroanatomical ontologies

| **Ontologies** | **Anatomical entities, in the increasing order of granularity** | | **Modeled**  **Ontological**  **Relations** | **Comments** |
| --- | --- | --- | --- | --- |
|  | **Lobar or Hemisphere** | **DKT atlas’s 100 ROIs** |  |  |
| Rosse and Mejino, 2003; Golbreich et al., 2006 | ✘ | ✘ | Part-of | A framework for representing the FMA model for human anatomy in W3C semantic web standards (Golbreich et al., 2006). Not brain specific (Rosse and Mejino, 2003; Golbreich et al., 2006). |
| Turner et al., 2010 | Partially | ✘ | Part-of | Includes gene entities. |
| Talos et al., 2008; Malhotra et al., 2014; French et al., 2015; Richardet et al., 2015; Gökdeniz et al., 2016 | Partially | Partially | Connectivity | Created symbolic neuroanatomy ontology of the motor system. Includes entities for motor nerves (Talos et al., 2008). Uses NLP text mining methods to establish ROI connectivity relationships and does not use W3C semantic web standards (Malhotra et al., 2014; French et al., 2015; Richardet et al., 2015; Gökdeniz et al., 2016). Includes animal species (French et al., 2015). First attempt at representing relevant knowledge on AD (Malhotra et al., 2014). |
| Kerwin et al., 2010 | ✔ | ✘ |  | Represents embryonic brain development during Carnegie stages. Includes gene entities. Does not use W3C semantic web standards. |
| Martin et al., 2001; Golbreich et al., 2003; Knublauch et al., 2004; Golbreich et al., 2005 | ✔ | ✘ | Part-of | Includes entities for other organs like the spine and digestive tract (Martin et al., 2001). |
| Hunter et al., 2003 | ✔ | Partially | Part-of | An ontology representing embryonic Carnegie stages. Not brain specific. |
| Nichols et al., 2014 | ✔ | Partially (DK atlas) aligned. | Connectivity & Part-of | Includes corticospinal entities. |
| Our Model | ✔ | ✔ | Part-of | Only includes brain-relevant concepts. Excludes functional connectivity relationships. Explicitly declares anatomical relations within the W3C semantic web standards. |

The check mark (✔) represents that a study models every anatomical concept at a given abstraction, ‘partially’ represents that a study models some anatomical concepts at a given abstraction, and the cross (✘) represents that a study models none of the anatomical concepts at a given abstraction. Part-of: structural anatomical membership, Connectivity: functional connectivity. FMA: Foundational Model of Anatomy, DKT: Desikan–Killiany–Tourville atlas of neuroanatomy, AD: Alzheimer's disease.

**References**

Boeker, M., Tudose, I., Hastings, J., Schober, D., Schulz, S., 2011. Unintended consequences of existential quantifications in biomedical ontologies. BMC Bioinform 12, 456.

Dumontier, M., Baker, C.J., Baran, J., Callahan, A., Chepelev, L., Cruz-Toledo, J., Del Rio, N.R., Duck, G., Furlong, L.I., Keath, N., Klassen, D., McCusker, J.P., Queralt-Rosinach, N., Samwald, M., Villanueva-Rosales, N., Wilkinson, M.D., Hoehndorf, R., 2014. The Semanticscience Integrated Ontology (SIO) for biomedical research and knowledge discovery. J Biomed Seman 5, 14.

French, L., Liu, P., Marais, O., Koreman, T., Tseng, L., Lai, A., Pavlidis, P., 2015. Text mining for neuroanatomy using WhiteText with an updated corpus and a new web application. Front Neuroinform 9, 13.

Gökdeniz, E., Özgür, A., Canbeyli, R., 2016. Automated neuroanatomical relation extraction: A linguistically motivated approach with a PVT connectivity graph case study. Front Neuroinform 10, 39.

Golbreich, C., Dameron, O., Bierlaire, O., Gibaud, B., 2005. What reasoning support for ontology and rules? the brain anatomy case study. Workshop on OWL Experiences and Directions, Nov 2005, Galway, Ireland.

Golbreich, C., Dameron, O., Gibaud, B., Burgun, A., 2003. Web Ontology Language Requirements w.r.t Expressiveness of Taxonomy and Axioms in Medicine, In: Goos, G., Hartmanis, J., van Leeuwen, J., Fensel, D., Sycara, K. and Mylopoulos, J. (Eds.), The Semantic Web - ISWC 2003. Springer Berlin Heidelberg, Berlin, Heidelberg, pp. 180–194.

Golbreich, C., Zhang, S., Bodenreider, O., 2006. The foundational model of anatomy in OWL: Experience and perspectives. Web Semantics (Online) 4, 181–195.

Hunter, A., Kaufman, M.H., McKay, A., Baldock, R., Simmen, M.W., Bard, J.B.L., 2003. An ontology of human developmental anatomy. J Anat 203, 347–355.

Jackson, R., Matentzoglu, N., Overton, J.A., Vita, R., Balhoff, J.P., Buttigieg, P.L., Carbon, S., Courtot, M., Diehl, A.D., Dooley, D.M., Duncan, W.D., Harris, N.L., Haendel, M.A., Lewis, S.E., Natale, D.A., Osumi-Sutherland, D., Ruttenberg, A., Schriml, L.M., Smith, B., Stoeckert, C.J., Vasilevsky, N.A., Walls, R.L., Zheng, J., Mungall, C.J., Peters, B., 2021. OBO Foundry in 2021: operationalizing open data principles to evaluate ontologies. Database (Oxford) 2021, baab069.

Kerwin, J., Yang, Y., Merchan, P., Sarma, S., Thompson, J., Wang, X., Sandoval, J., Puelles, L., Baldock, R., Lindsay, S., 2010. The HUDSEN Atlas: a three-dimensional (3D) spatial framework for studying gene expression in the developing human brain. J Anat 217, 289–299.

Knublauch, H., Dameron, O., Musen, M., 2004. Weaving the biomedical Semantic Web with the Prote'ge' OWL Plugin, 39 pp.

Malhotra, A., Younesi, E., Gündel, M., Müller, B., Heneka, M.T., Hofmann-Apitius, M., 2014. ADO: a disease ontology representing the domain knowledge specific to Alzheimer's disease. Alzheimers Dement 10, 238–246.

Martin, R., Mejino, J., Bowden, D., Brinkley, J., Rosse, C., 2001. Foundational model of neuroanatomy: implications for the Human Brain Project. Proceedings / AMIA … Annual Symposium. AMIA Symposium 2001, 438–442.

Mejino, J., Rubin, D., Brinkley, J., 2008. FMA-RadLex: An Application Ontology of Radiological Anatomy derived from the Foundational Model of Anatomy Reference Ontology. AMIA … Annual Symposium proceedings / AMIA Symposium. AMIA Symposium 2008, 465–469.

Nichols, B.N., Mejino, J.L., Detwiler, L.T., Nilsen, T.T., Martone, M.E., Turner, J.A., Rubin, D.L., Brinkley, J.F., 2014. Neuroanatomical domain of the foundational model of anatomy ontology. J Biomed Semant 5, 1.

Richardet, R., Chappelier, J.-C., Telefont, M., Hill, S., 2015. Large-scale extraction of brain connectivity from the neuroscientific literature. Bioinformatics (Oxford) 31, 1640–1647.

Rosse, C., Mejino, J.L.V., 2003. A reference ontology for biomedical informatics: the Foundational Model of Anatomy. J Biomed Inform 36, 478–500.

Talos, I.-F., Rubin, D.L., Halle, M., Musen, M., Kikinis, R., 2008. A prototype symbolic model of canonical functional neuroanatomy of the motor system. J Biomed Inform 41, 251–263.

Turner, J.A., Mejino, J.L.V., Brinkley, J.F., Detwiler, L.T., Lee, H.J., Martone, M.E., Rubin, D.L., 2010. Application of neuroanatomical ontologies for neuroimaging data annotation. Front Neuroinform 4: 10.

**Supplemental Material 3. Aggregating pathology at the parent nodes**

We applied the metric w-score, which is akin to z-scores for quantifying deviation from the expected healthy control values. If *y* is the estimated ROI measure obtained from the segmentation tool, and *e_controls_* is the residuals of the healthy controls, then, w-score is defined as.

$W=\frac{y - ŷ}{Std(e_{controls} )}$ (1)

We modeled a hierarchical relationship amongst all ROIs from segmentation tool which were defined as child nodes, and introduced several parent nodes at higher levels according to anatomical abstractions (e.g., lobes and hemispheres). Notably, we intentionally modeled this ontology as a strict taxonomy. That means, we require the ontology 1) to be a tree structure and 2) all ROIs from the segmentation tool to be included as leaf nodes in the tree. Additionally, we assumed that all parent node measures (such as volume and cortical thickness) could be directly calculated based on the information of their direct child nodes. This design allows recursive processing of the parent-ROIs without explicitly training the linear regression models for the parent levels.

To simplify the formal notation, we describe a scenario where the parent ROI had only two child-ROIs. However, the inferences drawn hold true for a parent ROI with an arbitrary number of child regions. Given y: true ROI measure, ŷ: predicted ROI measure, p: parent-ROI, c_i_: i^th^ child-ROI, ɑ: vector of coefficients of a linear regression model, β: intercept of the linear regression model, e: error or residual, CV: vector of confounding variables [age, sex, MRI field strength, brain size]. Where:

$\hat{y}_{c1}= \alpha_{c1}*CV+ \beta_{c1}$ (2)

$\hat{y}_{c2}= \alpha_{c2}*CV+ \beta_{c2}$ (3)

When $y_{p}= y_{c1}+ y_{c2}$ (4)

and $\hat{y}_{p}= \hat{y}_{c1}+ \hat{y}_{c2}$ (5)

it follows that, for a specific set of confounding variables, i.e., for each specific data subject:

$\hat{y}_{p}= \alpha_{c1}*CV+ \beta_{c1} +\alpha_{c2}*CV+ \beta_{c2}$ (6)

$\hat{y}_{p}= {(\alpha}_{c1}+\alpha_{c2})*CV+ {(\beta}_{c1}+\beta_{c2})$ (7)

Eq(7) shows that, for each parent-ROI, one could additively infer the linear model from its child-ROI, which controls for confounding variables and predicts the expected volume measurements.

Subtracting eq(5) from eq(4), we obtain the residual for the parent node:

$y_{p}-\hat{y}_{p}= {(y}_{c1}+ y_{c2})-(\hat{y}_{c1}+ \hat{y}_{c2})$ (8)

$y_{p}-\hat{y}_{p}= {(y}_{c1}-\hat{y}_{c1})+(y_{c2}-\hat{y}_{c2})$ (9)

$e_{p} = e_{c1}+ e_{c2}$ (10)

Eq(10) shows that for a parent-ROI one could additively infer the residual term (also known as the error) of the LR model from its constituent child-ROI’s residual term. Using eq(7) and eq(10) and the neuroanatomical ontology described in the previous section, one can efficiently quantify volumetric deviations (w-score) from healthy controls at all abstraction levels. This includes ROIs that were not direct outputs of the segmentation tool, i.e., the parent-ROIs.

It should be noted that this conceptual modeling of additive linear regression models for implicitly deriving w-scores at all ROI levels, only holds when eq(4) holds. The aggregation of the cortical thickness requires weighted averages with region’s surface area, and does not strictly adhere to the eq(4). For measures such as these, we skip the additive framework and train a linear regression model for each region separately.

Furthermore, there are two underlying assumptions. First, each child-ROI must have one and only one parent-ROI. Second, ROIs must be disjoint at the voxel level, i.e., post segmentation, each voxel inside brain volume must get exactly one label from the atlas. These assumptions may be violated for probabilistic or functional neuroimaging atlases, where one voxel could be a member of multiple ROIs or functional networks. However, these assumptions are well suitable for the common application of neuroanatomical volumetric analyses.

**Supplemental Material 4. ADNI patient ID 6849’s Cortical Thickness Summary Plot**

**Supplemental Figure 2.** Single-subject *average cortical thickness* sunburst chart for the ADNI Alzheimer’s patient ID 6849 with mild dementia. The regions for which FastSurfer does not produce cortical thickness measures were grayed-out, for example subcortical regions, white matter and cerebrospinal fluid compartments.


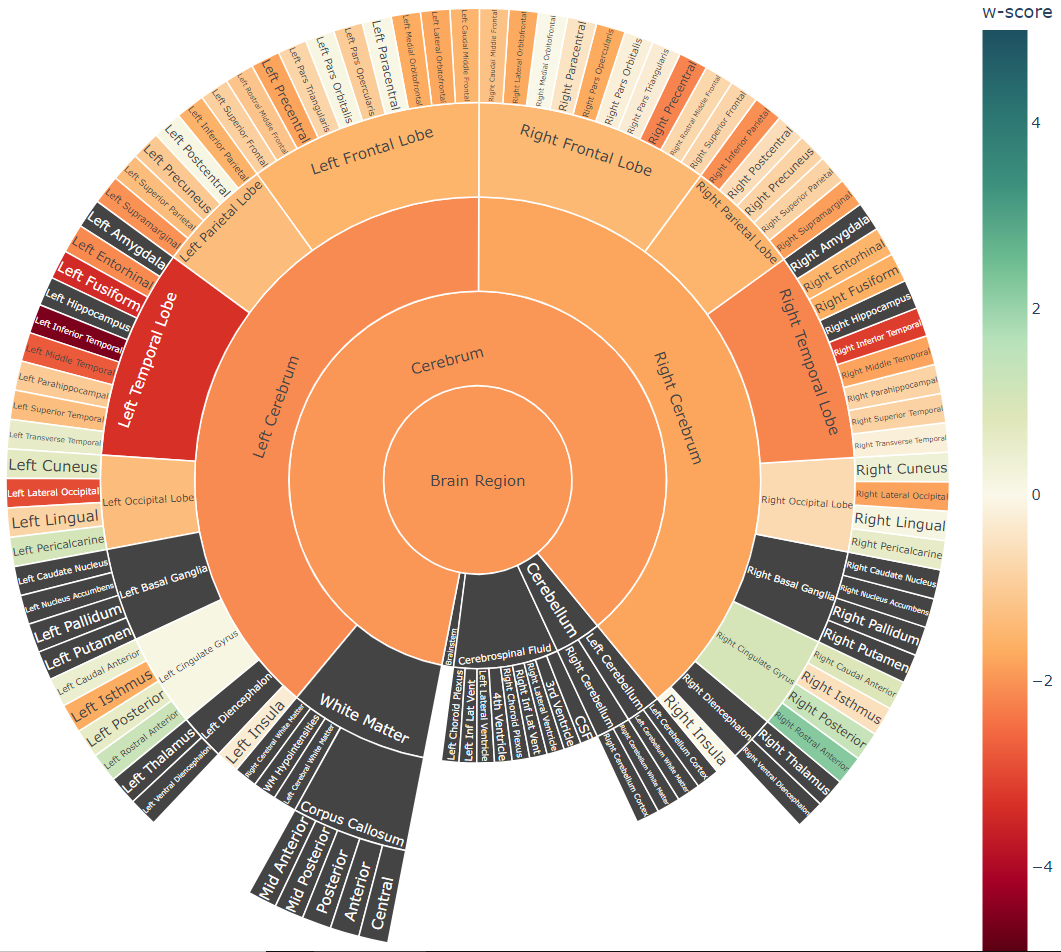


**Supplemental Material 5. ADNI patient ID 6849’s maximum pathology Summary Plot**

**Supplemental Figure 3.** Single-subject sunburst chart for the region-wise *maximum pathology* obtained from both *volume* and *cortical thickness,* for the ADNI Alzheimer’s patient ID 6849.


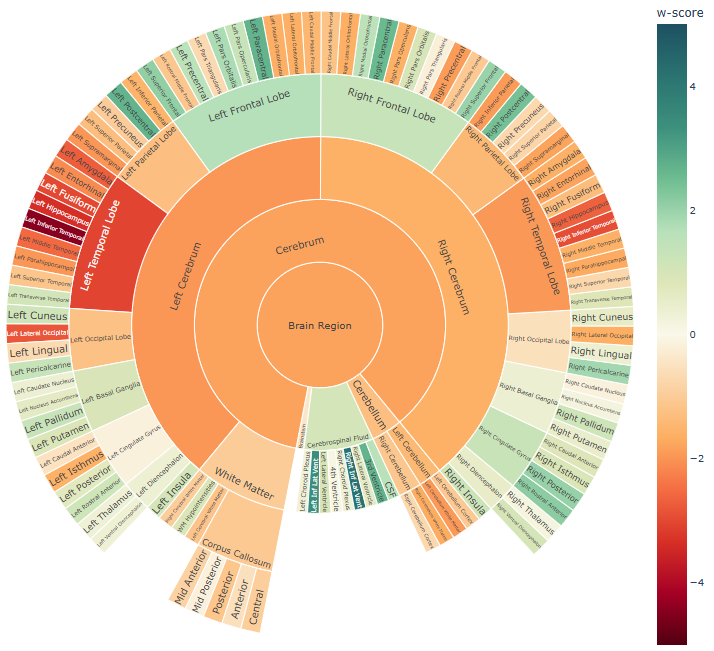


**Supplemental Material 6. Volumetric Summary plots by cohorts**

**Supplemental Figure 4.** Summary plots, illustrating mean volume w-scores across ROIs at lobe level, for the healthy control (HC) subjects grouped by data cohorts: (a) ADNI2, (b) ADNI3, (c) AIBL, (d) DELCODE, (e) DESCRIBE, (f) EDSD and (g) NIFD.

**Healthy Controls (HC)**


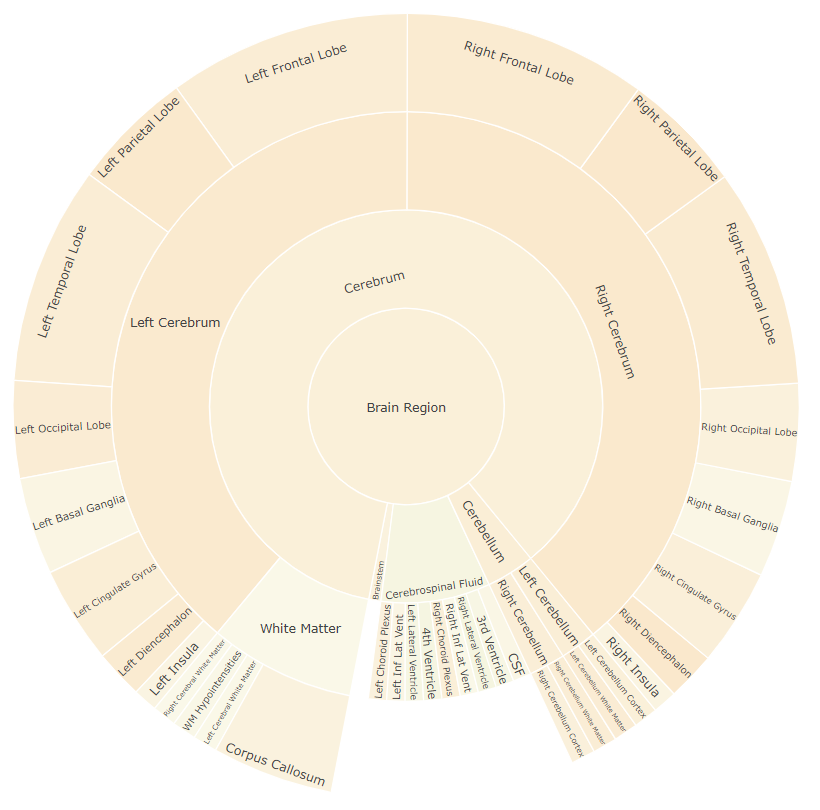

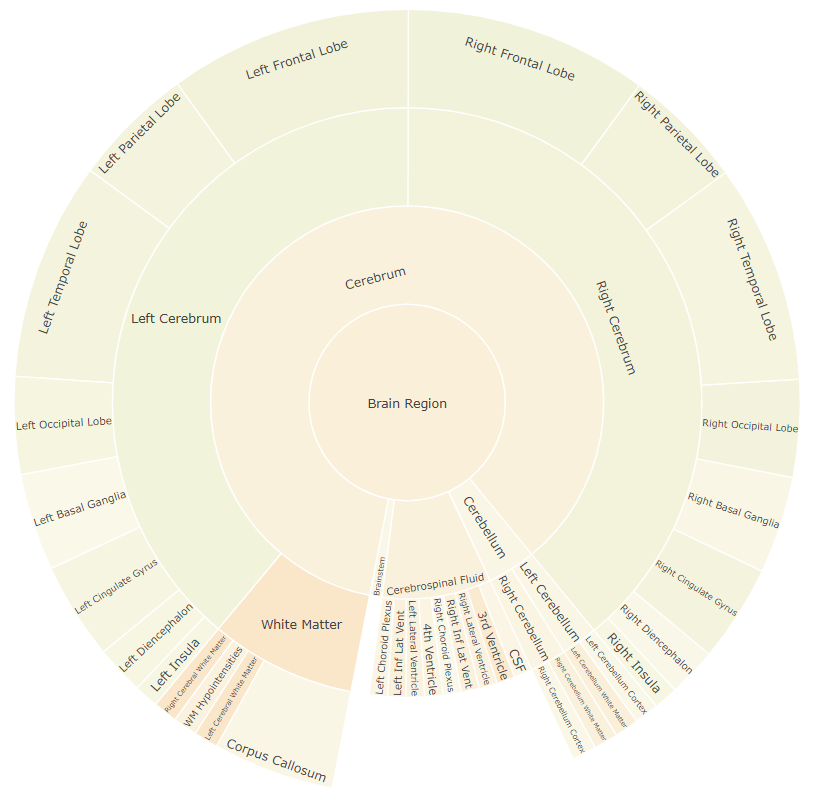

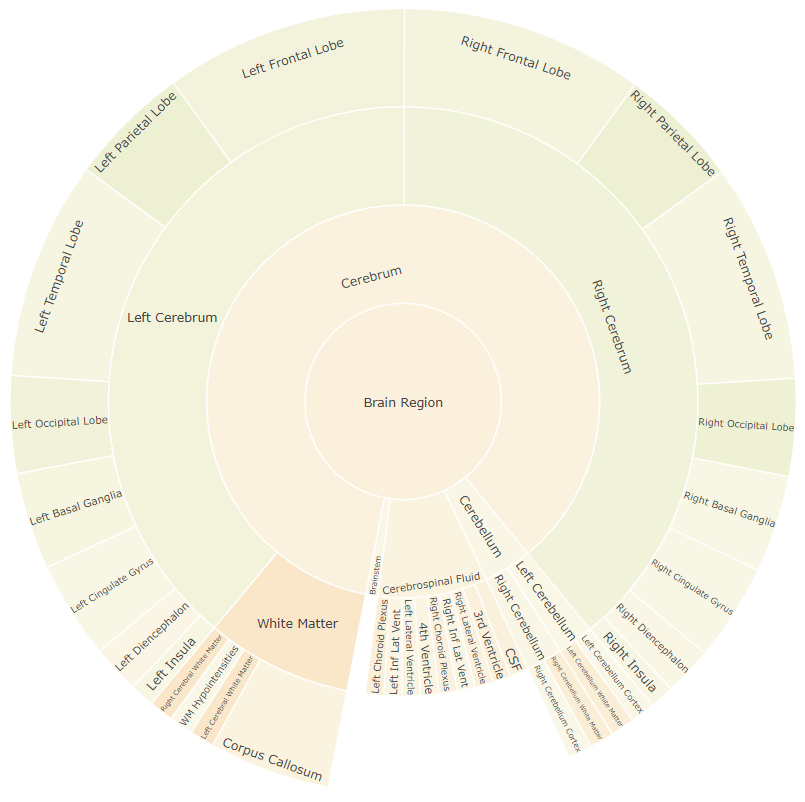

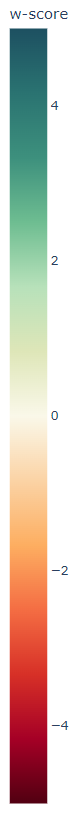


        (a) ADNI2               (b) ADNI3           (c) AIBL


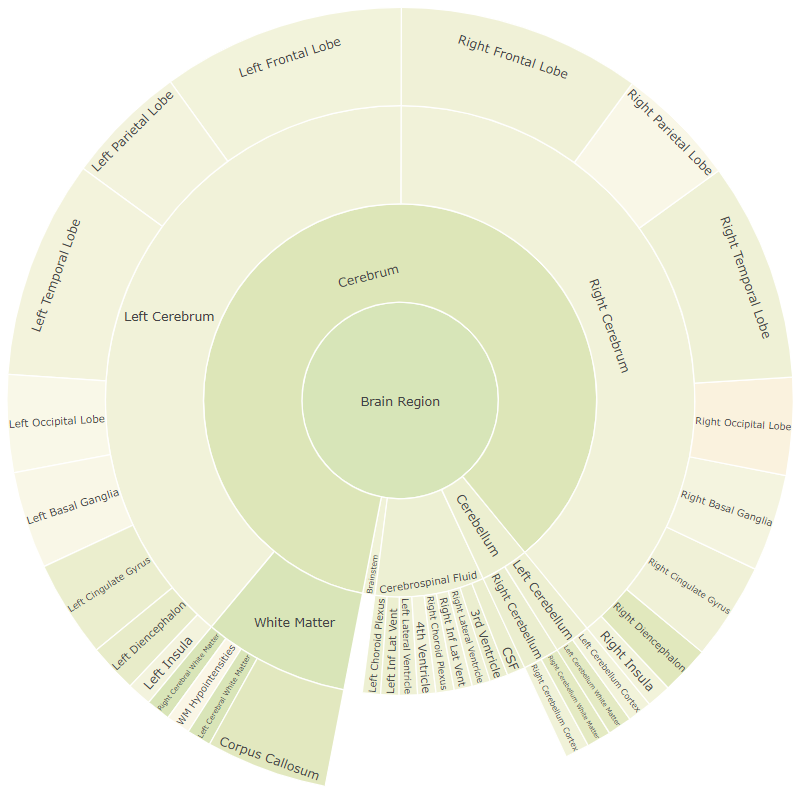

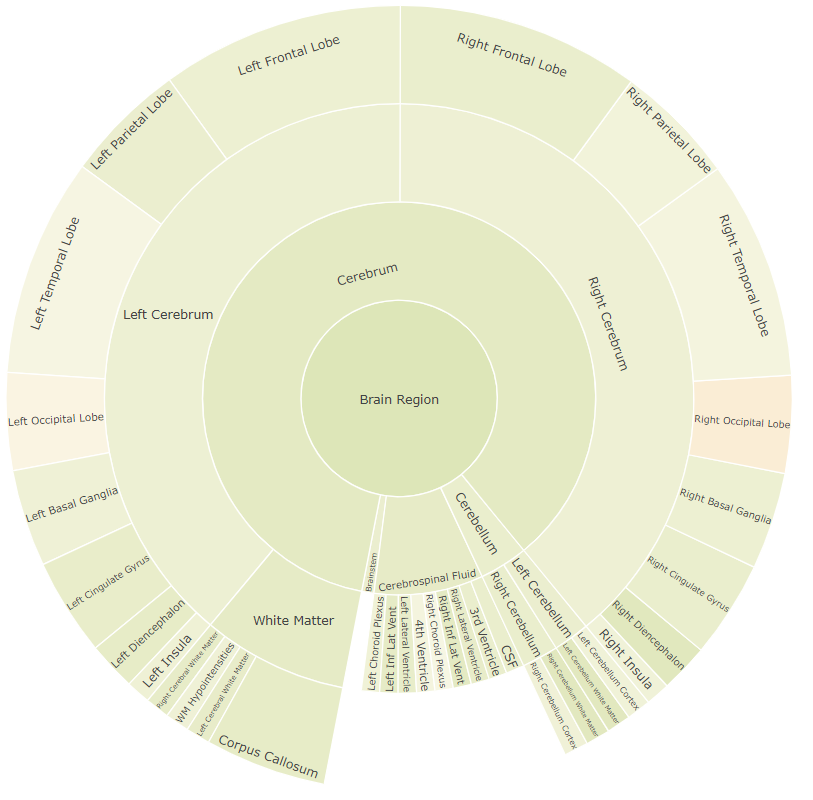

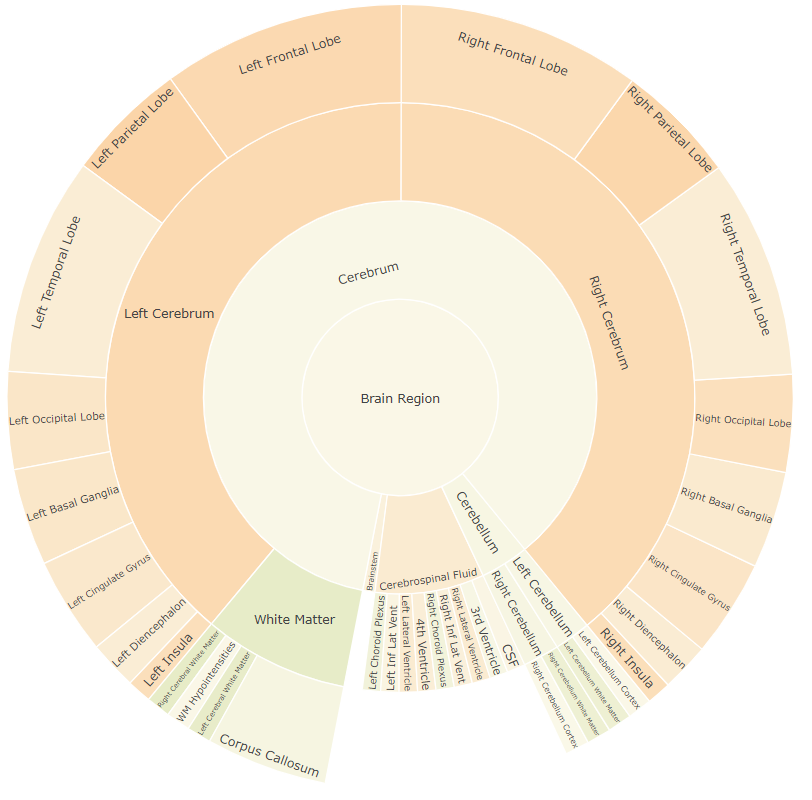


            (d) DELCODE (e) DESCRIBE           (f) EDSD


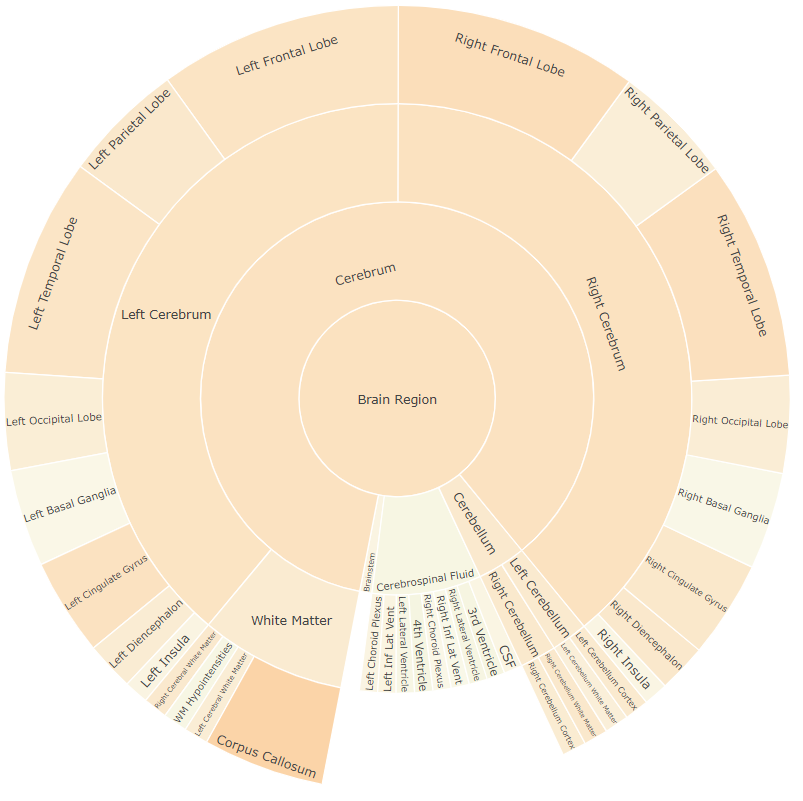


(g) NIFD

**Supplemental Figure 5.** Summary plots, illustrating mean volume w-scores across ROIs at lobe level, for the people with mild cognitive impairment (MCI) grouped by data cohorts - (a) ADNI2, (b) ADNI3, (c) AIBL, (d) DELCODE, and (e) EDSD.

**Mild Cognitive Impairment (MCI)**


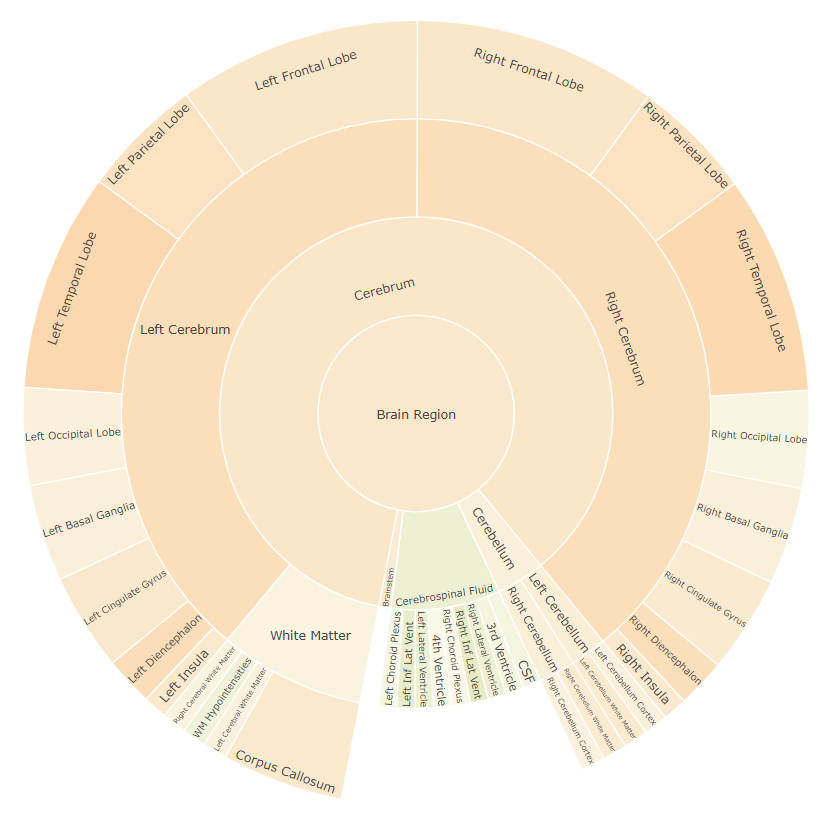

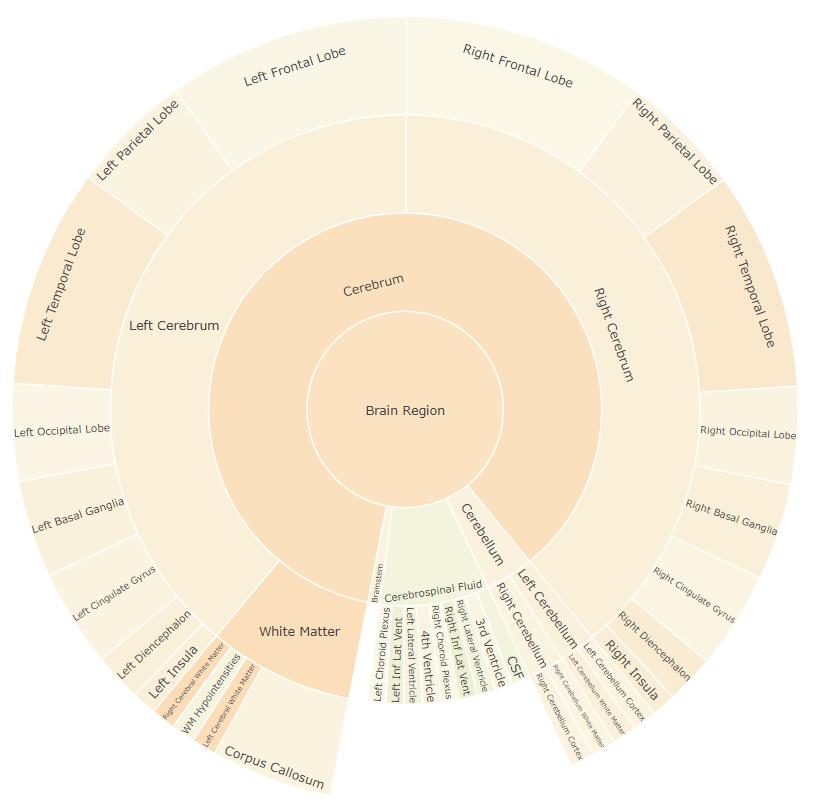

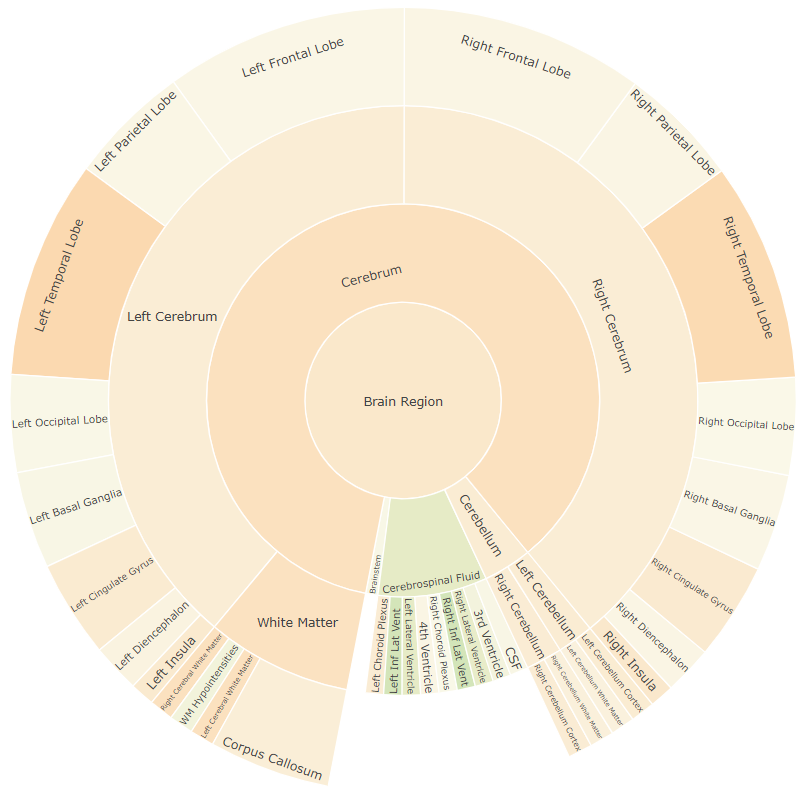

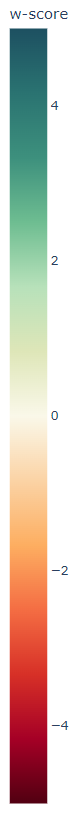


              (a) ADNI2            (b) ADNI3           (c) AIBL


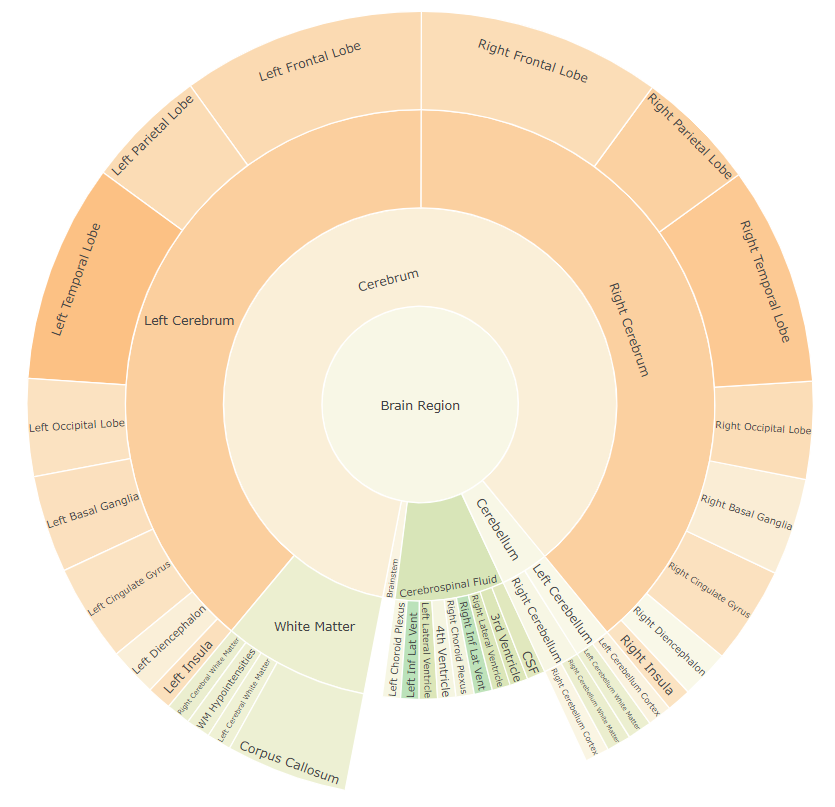

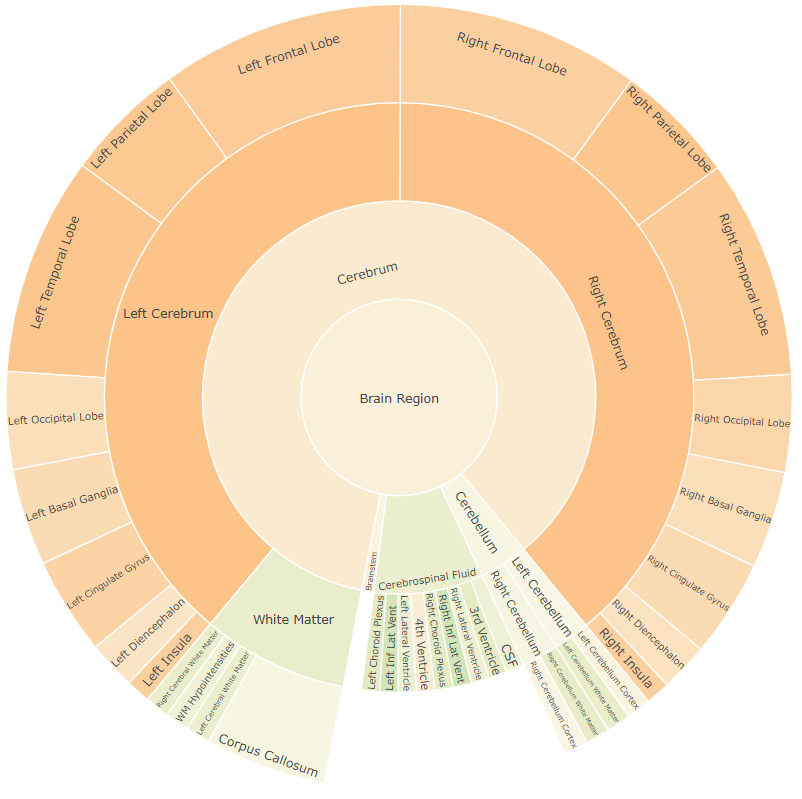


                       (d) DELCODE     (e) EDSD

**Supplemental Figure 6.** Summary plots, illustrating mean volume w-scores across ROIs at lobe level, for the Alzheimer's disease (AD) subjects grouped by data cohorts - (a) ADNI2, (b) ADNI3, (c) AIBL, (d) DELCODE, and (e) EDSD.

**Dementia due to Alzheimer’s Disease (AD)**


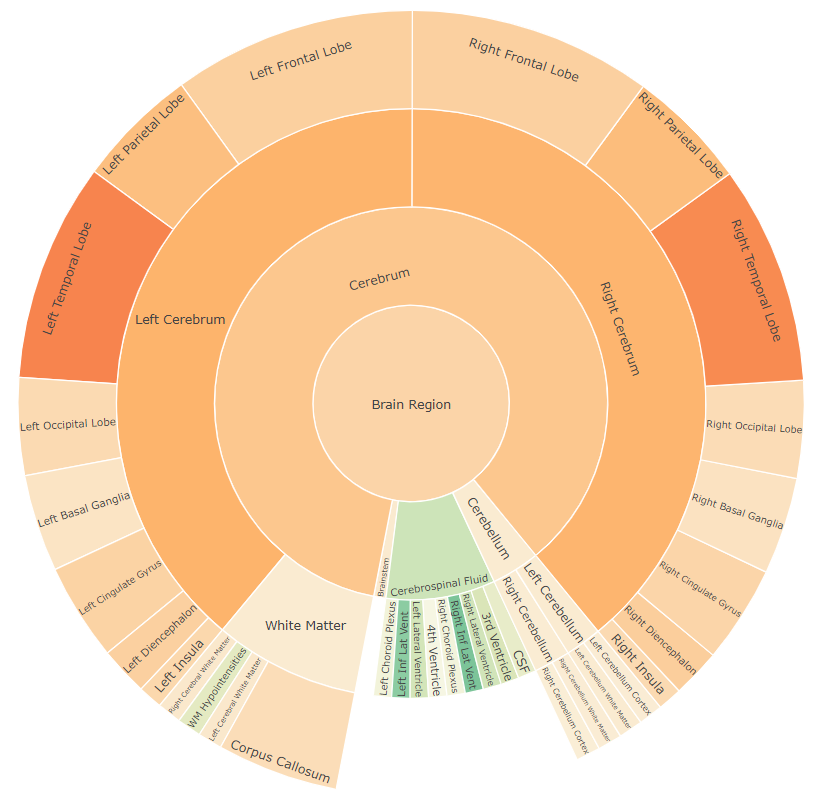

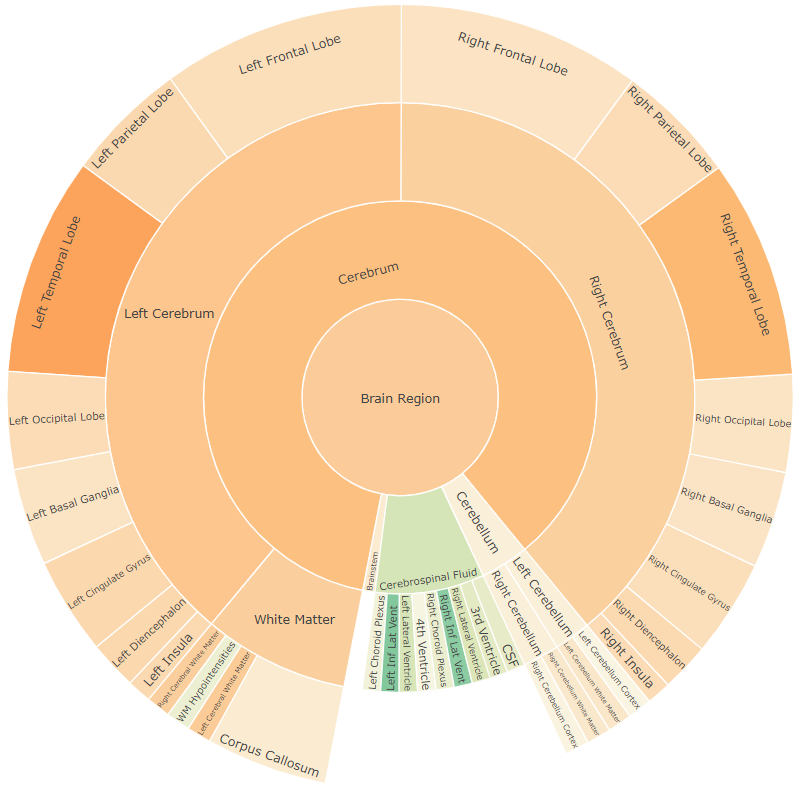

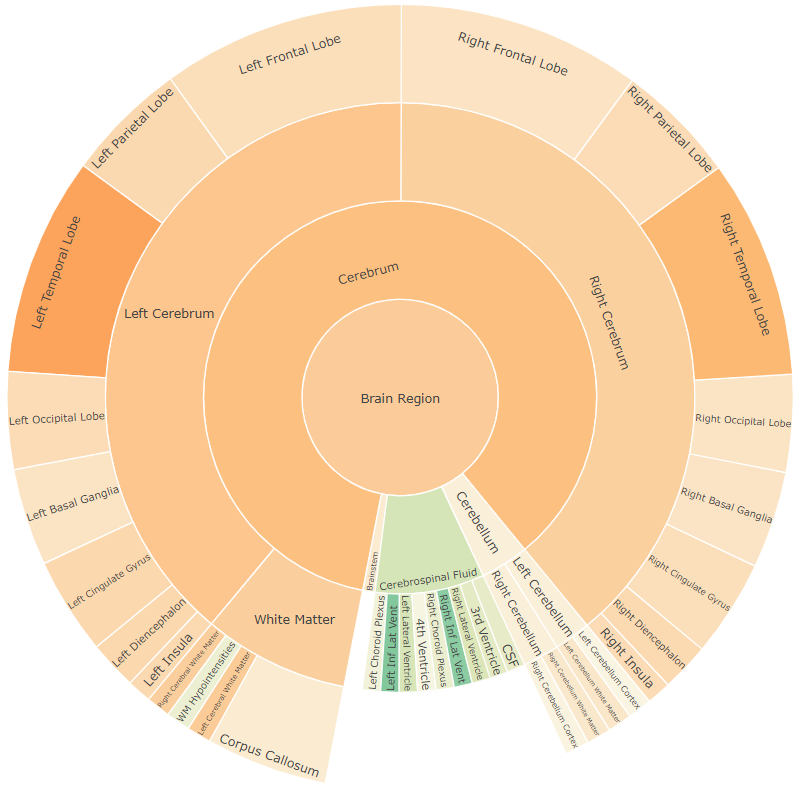

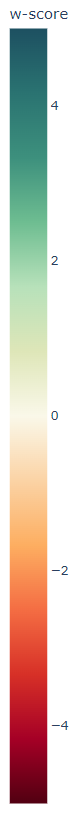


               (a) ADNI2              (b) ADNI3           (c) AIBL


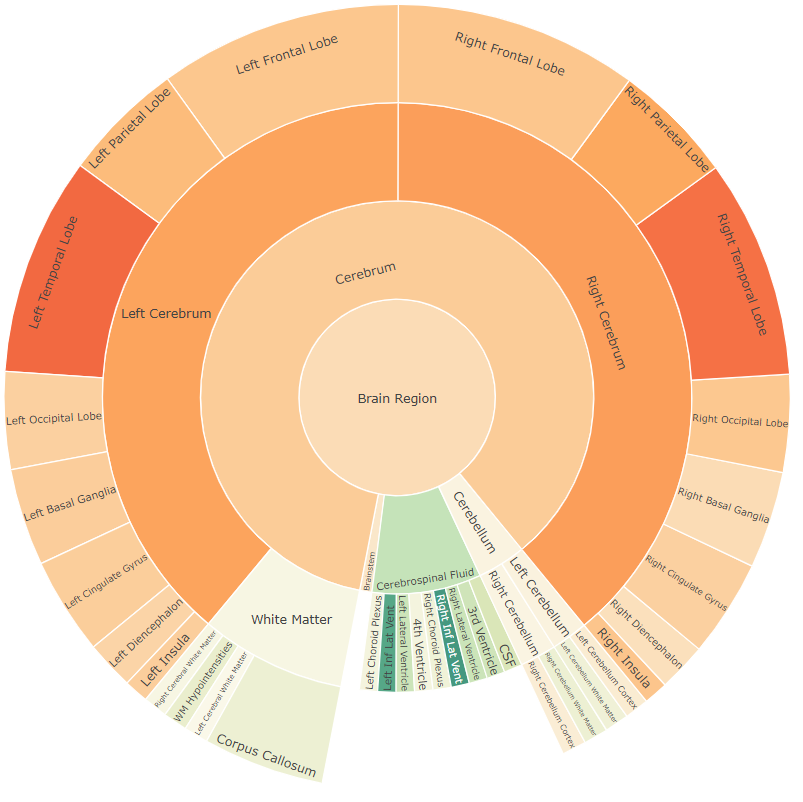

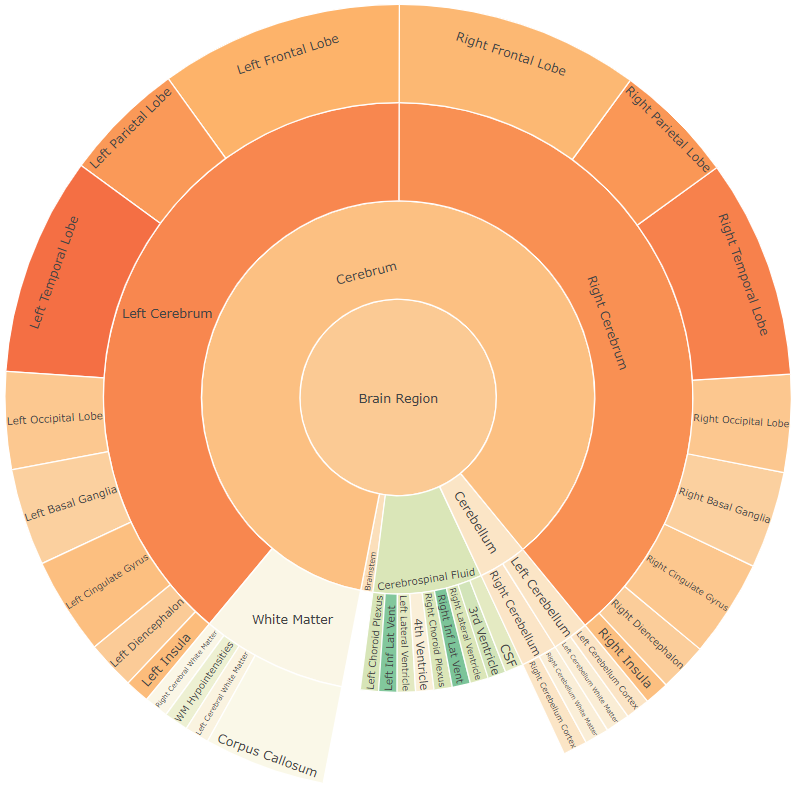


(d) DELCODE           (e) EDSD

**Supplemental Figure 7.** Summary plots, illustrating mean volume w-scores across ROIs at lobe level, for the behavioral variant frontotemporal dementia (bvFTD) subjects grouped by data cohorts - (a) DESCRIBE, and (b) NIFD.

**Behavioral Variant of Frontotemporal Dementia (bvFTD)**


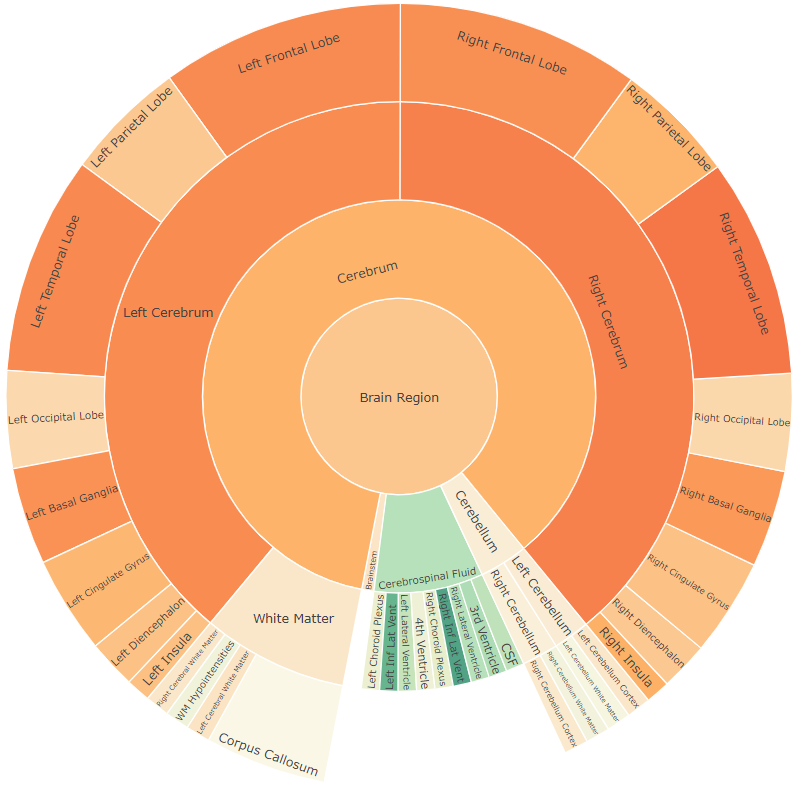

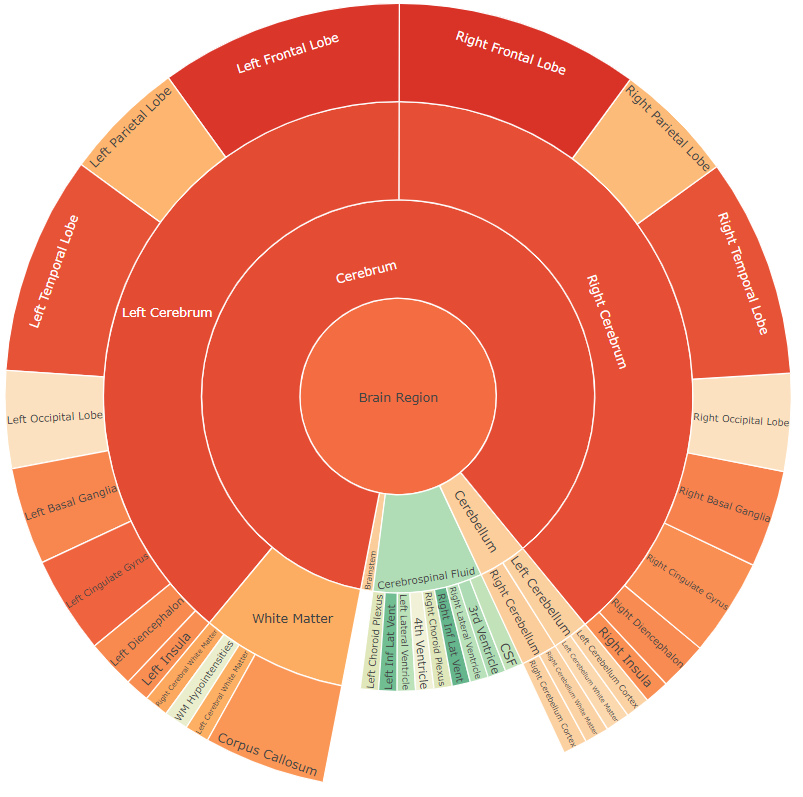

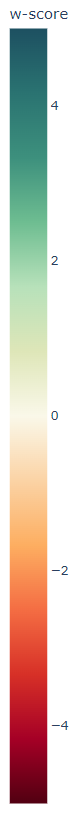


                      (a) DESCRIBE                                     (b) NIFD

**Supplemental Material 7. Cortical thickness summary plots**

**Supplemental Figure 8.** Sunburst summary plots illustrating *mean cortical thickness* w-scores across ROIs at the lobe level, highlighting differences between disease stages of AD, and dementia types. HC: healthy controls, MCI: mild cognitive impairment, AD: dementia due to Alzheimer's disease, bvFTD: behavioral variant of frontotemporal dementia. Please refer to the online version of the plots for a more detailed visualization. The regions for which FastSurfer does not produce cortical thickness measures were grayed-out.


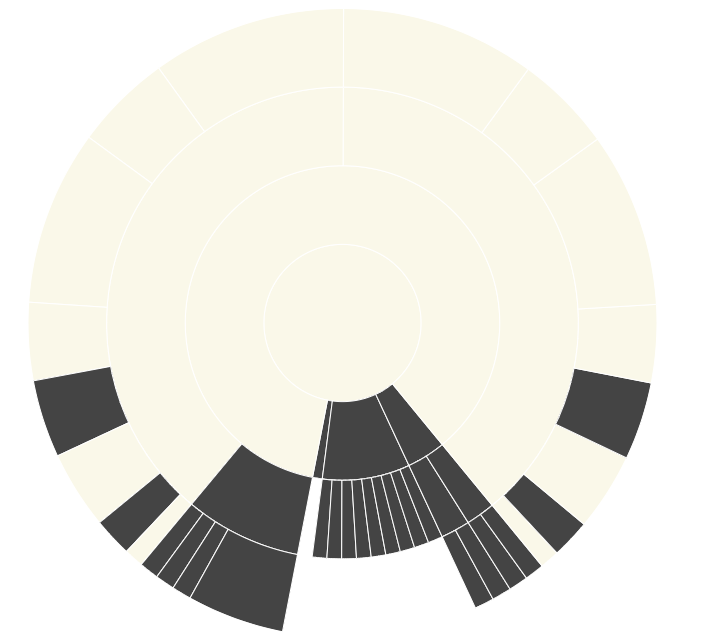

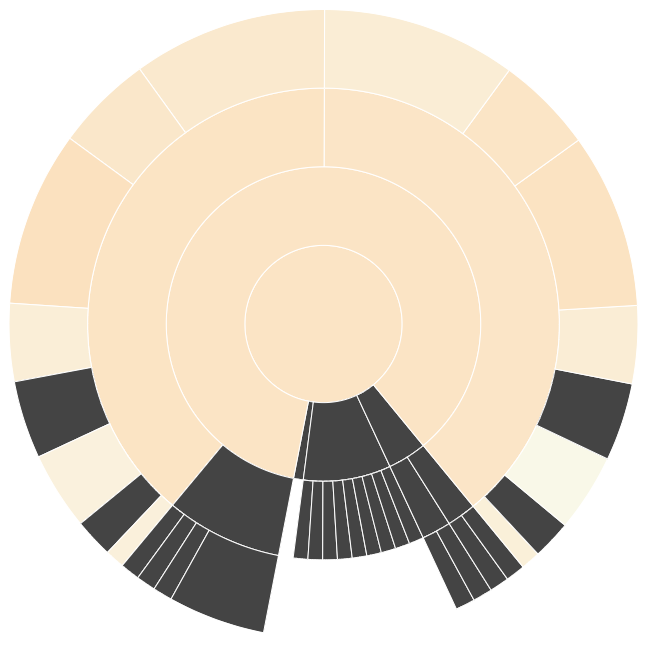


(a) HC (b) MCI


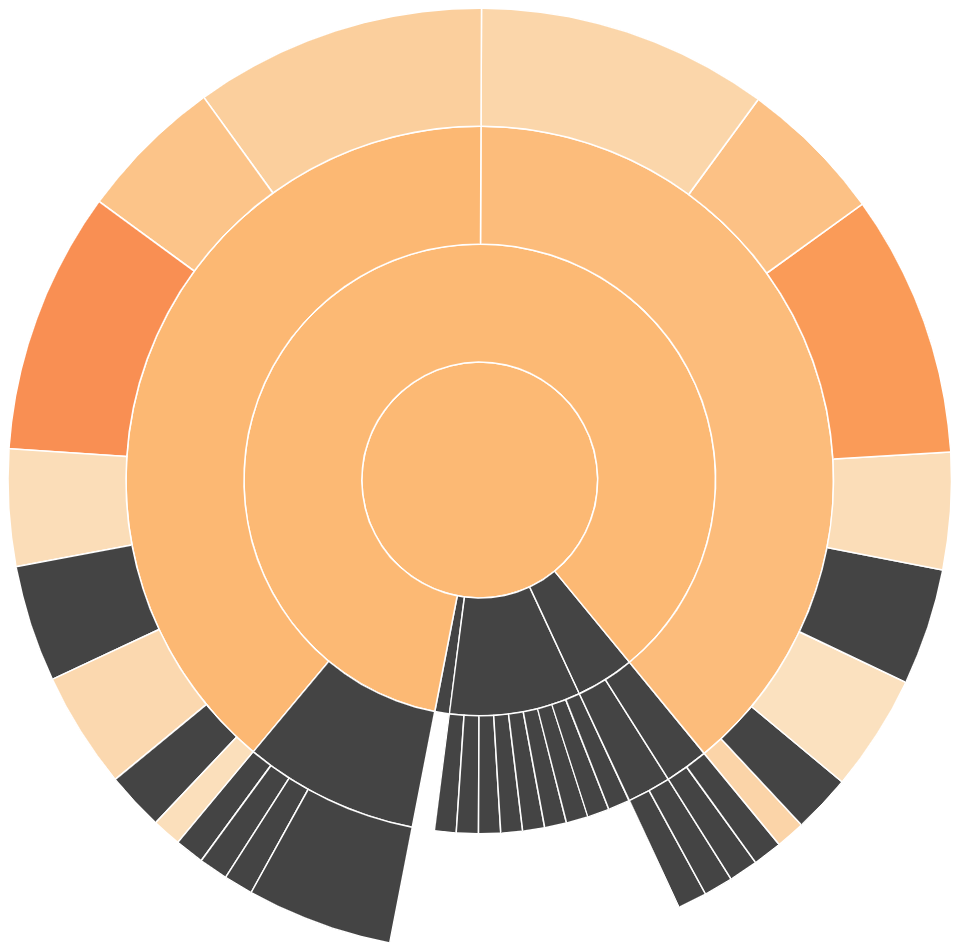

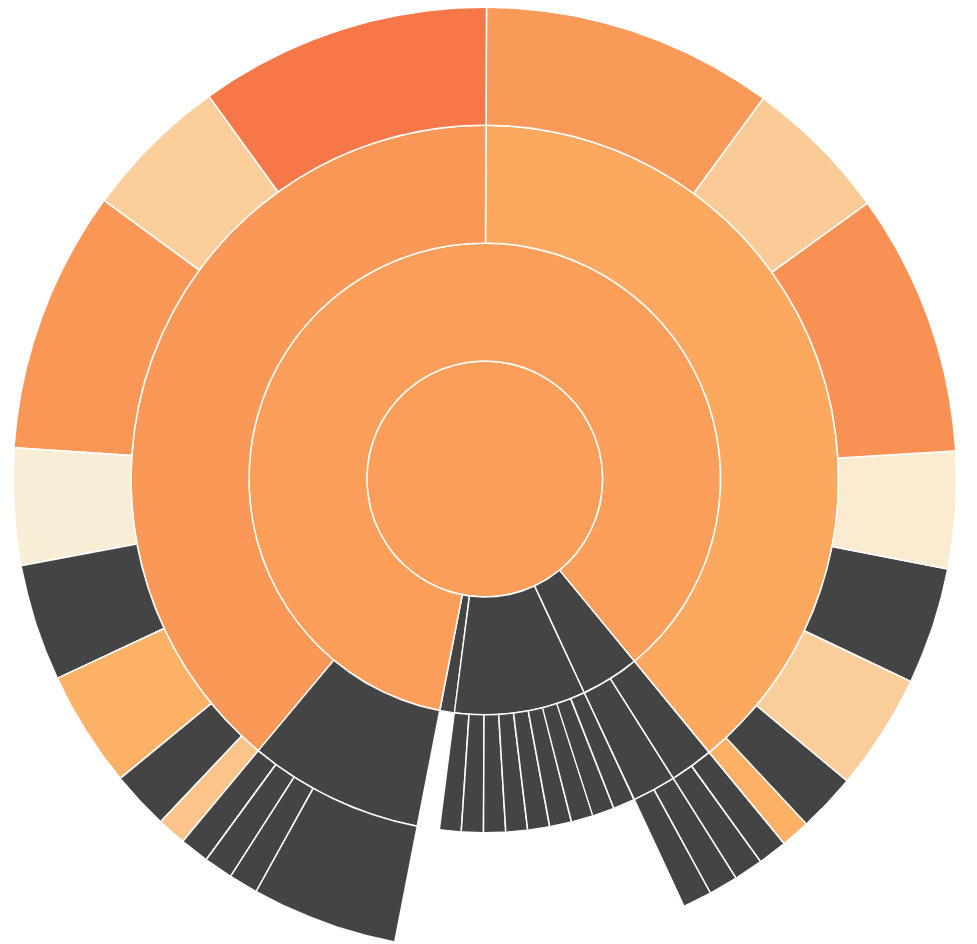


(c) AD (d) bvFTD

**Supplemental Material 8. Cosine similarity metric for disease type separation**

In the following subsection, we evaluate the cosine similarity metric, over w-score profiles (i.e., the volumetric and cortical thickness deviation quantification from normal levels), for mapping people with dementia to specific neurodegenerative diseases. More specifically, for the group separation task where we only consider the subjects diagnosed with Alzheimer's disease (AD) (N=463) and behavioral variant frontotemporal dementia (bvFTD) (N=126), we want to quantify if the cosine similarity metric can reliably distinguish between the two disease types. It is of interest for us to understand the amount of group information captured by the w-score profile cosine similarity. For each sample of the AD and bvFTD group, we calculate the cosine similarity between the sample and the mean w-score profiles for the healthy control (HC), AD, and bvFTD group. We also establish a metric *‘disease similarity index’*, defined as:

$DiseaseSimilarityIndex(x)= cos(x,AD_{w-score})-cos(x, bvFTD_{w-score})$ (A.8.1)

Where, $x$ is a sample’s w-score profile, i.e., the vector of w-scores for each ROI in the ontology, cos(.) is the cosine similarity metric, $AD_{w-score}$ is the mean w-score profile for the AD group, and $bvFTD_{w-score}$ is the mean w-score profile for the bvFTD group.

In Supplemental Figure 9, we illustrate the receiver operating characteristic (ROC) curve and the associated area under the curve (AUC) metric of 0.89 for the disease similarity index, with AD being the positive class. In an extended 10-fold cross validation analysis, the ROC-AUC was found to be 0.88±0.05.

To understand the group separability capacity of the disease similarity index, we threshold the disease similarity index at the zero point (the red line in Supplemental Figure 11), i.e., a point at which for a sample both diseased types are equally likely. Supplemental Figure 10 illustrates the confusion matrix associated with this zero point. Assuming this threshold to be the decision boundary we found the further evaluation metrics from the 10-fold cross validation analysis to be - balanced accuracy: 0.8±0.04, sensitivity: 0.75±0.06, specificity: 0.84±0.04, positive predictive value: 0.57±0.09, negative predictive value: 0.93±0.02, and the F1-score: 0.65±0.07. Supplemental Table 3 details the evaluation metrics found from each fold of the cross-validation analysis of the disease similarity index.

This extended analysis of the disease similarity index implies a good group separation capacity of the cosine similarity metric calculated on volumetric and cortical thickness w-scores profiles, for the AD-vs-bvFTD group separation task. The 10-fold cross validation analysis also demonstrates the relative robustness and stability of the disease similarity index. However, one should exert care while using the disease similarity index for the mild cognitive impairment (MCI) patients, as they have an intermediate pathological presentation between the healthy stage and the AD stage, and thus get mixed results from the disease similarity index (see Supplemental Figure 11).

**Supplemental Figure 9.** The receiver operating characteristic (ROC) curve for the disease similarity index. AD represents the positive class, bvFTD the negative class.


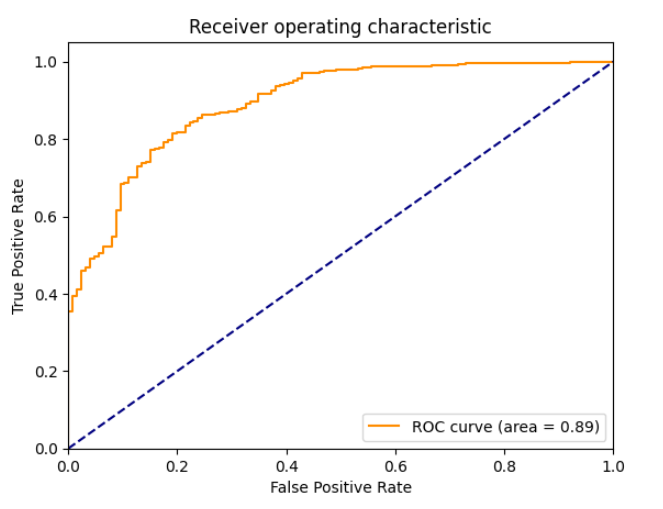


**Supplemental Table 3.** Evaluation metrics from 10-fold cross validation analysis of disease similarity index. auc: Area under the (ROC) curve, bacc: balanced accuracy, sen: sensitivity, spec: specificity, ppv: positive predictive values, npv: negative predictive values, f1: balanced F-score, and acc: (simple) accuracy.

| **Fold** | **auc** | **bacc** | **sen** | **spec** | **ppv** | **npv** | **f1** | **acc** |
| --- | --- | --- | --- | --- | --- | --- | --- | --- |
| 1 | 0.79 | 0.75 | 0.69 | 0.81 | 0.50 | 0.90 | 0.58 | 0.78 |
| 2 | 0.92 | 0.87 | 0.85 | 0.89 | 0.69 | 0.95 | 0.76 | 0.88 |
| 3 | 0.82 | 0.74 | 0.67 | 0.81 | 0.47 | 0.90 | 0.55 | 0.78 |
| 4 | 0.87 | 0.77 | 0.75 | 0.79 | 0.47 | 0.93 | 0.58 | 0.78 |
| 5 | 0.87 | 0.79 | 0.75 | 0.83 | 0.53 | 0.93 | 0.62 | 0.81 |
| 6 | 0.87 | 0.81 | 0.77 | 0.85 | 0.59 | 0.93 | 0.67 | 0.83 |
| 7 | 0.96 | 0.83 | 0.77 | 0.89 | 0.67 | 0.93 | 0.71 | 0.86 |
| 8 | 0.92 | 0.83 | 0.85 | 0.82 | 0.58 | 0.95 | 0.69 | 0.83 |
| 9 | 0.91 | 0.83 | 0.75 | 0.91 | 0.69 | 0.93 | 0.72 | 0.88 |
| 10 | 0.90 | 0.76 | 0.69 | 0.83 | 0.53 | 0.90 | 0.60 | 0.80 |
| Mean | 0.88 | 0.80 | 0.75 | 0.84 | 0.57 | 0.93 | 0.65 | 0.82 |
| Std.Dev. | 0.05 | 0.04 | 0.06 | 0.04 | 0.09 | 0.02 | 0.07 | 0.04 |

**Supplemental Figure 10.** The confusion matrix for the disease similarity index, thresholded at the zero point of DSI.


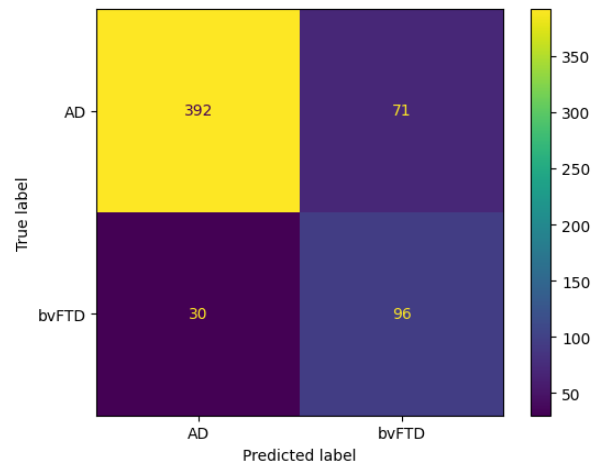


**Supplemental Figure 11.** The Disease Similarity Index (DSI) violin plot for different disease diagnosis groups - AD: Alzheimer's disease, MCI: Mild Cognitive Impairment or bvFTD: Behavioral variant of frontotemporal dementia. A red horizontal line marks the threshold at 0 (i.e., decision boundary) for separating the diseased types.


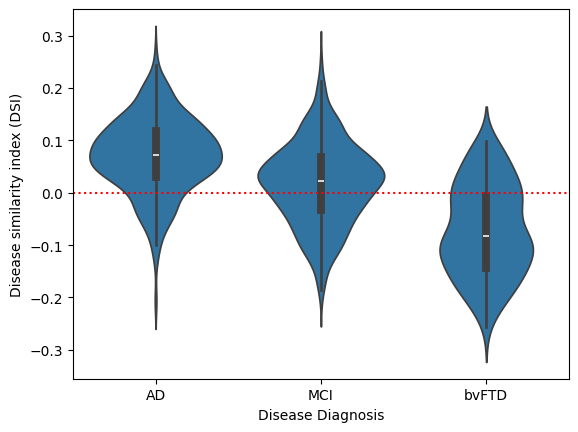

Supplement: sj-docx-1-alz-10.1177_13872877251331222 - Supplemental material for A computational ontology framework for the synthesis of multi-level pathology reports from brain MRI scans [file sj-docx-1-alz-10.1177_13872877251331222.docx]
